# Oligomeric (Salen)Mn(III) Complexes Featuring Tartrate Linkers Immobilized over Layered Double Hydroxide for Catalytically Asymmetric Epoxidation of Unfunctionalized Olefins

**DOI:** 10.3390/ma13214860

**Published:** 2020-10-29

**Authors:** Yihong Jia, Asma A. ALOthman, Rui Liang, Xiaoyong Li, Weiyi Ouyang, Xiangdong Wang, Yong Wu, Sameh M. Osman, Zhaorui Li, Min Gao, Yang Sun

**Affiliations:** 1 Department of Applied Chemistry, School of Science, Xi’an Jiaotong University, No. 28, Xianning West Road, Xi’an 710049, China; jiayihong@stu.xjtu.edu.cn (Y.J.); lixy6658@mail.xjtu.edu.cn (X.L.); weyi.ouyang@gmail.com (W.O.); wang90xd@163.com (X.W.); specwy@mail.xjtu.edu.cn (Y.W.); 2 Department of Chemistry, College of Science, King Saud University, P.O. Box 2455, Riyadh 11451, Saudi Arabia; aaalothman@ksu.edu.sa (A.A.A.); smahmoud@ksu.edu.sa (S.M.O.); 3 School of Resources and Environment, Anhui Agriculture University, No. 130, Changjiang West Road, Hefei 230036, China; liangrui@ahau.edu.cn; 4 College of Materials Science and Engineering, Xi’an University of Science and Technology, No. 48, Shaangu Avenue, Lintong District, Xi’an 710600, China; lzr981013@163.com

**Keywords:** (tartrate-salen)Mn(III), immobilization, layered double hydroxide, asymmetric epoxidation, enantioselectivity

## Abstract

A series of oligomeric (salen)Mn(III) complexes featuring tartrate linkers were prepared and immobilized over layered double hydroxide, and then used as catalysts for asymmetric epoxidation of unfunctionalized olefins. Comprehensive characterizations including ^1^H NMR, FT-IR, UV-Vis, elemental analysis, GPC, and ICP-AES were used to illustrate structures of oligomeric (salen)Mn(III) complexes, while powdered XRD, nitrogen physisorption, together with XPS studies provided further details to detect structures of heterogeneous catalysts. Interestingly, scanning electron microscopy found an interesting morphology change during modification of layered supporting material. Catalytic experiments indicated that configuration of major epoxide products was determined by salen chirality more than that of tartrate linker, but enantioselectivity (e.e. values) could be enhanced when tartrate and salen showed identical chiral configurations. Furthermore, the (*R,R*)-salen moieties linked with (*R,R*)-tartrate spacers usually offered higher enantioselectivity compared to other combinations. Lastly, Zn(II)/Al(III) layered double hydroxide played as a rigid supporting material in catalysis, showing positive chiral induction and high recycling potential in catalytic reactions.

## 1. Introduction

Synthesis of enantiomerically pure epoxides with high efficiency was of great importance and had attracted massive attentions in the fields of pharmaceutical chemistry and material science [1]. The design of efficient and effective catalysts was significant in the asymmetric epoxidation of alkenes to achieve high synthetic availability, optical purity, and atom economy, while addressing corresponding environmental concerns would give more opportunities to industrialization [2,3].

In this regard, Sharpless and co-workers established a Ti(IV)/tartrate system that afforded high enantioselectivities in asymmetric epoxidation of functionalized alkenes like allylic alcohols [4]. Later, chiral (salen)Mn(III) complexes were developed by Jacobsen [5] and Katsuki [6] as highly efficient epoxidation catalysts for transformations of unfunctionalized alkenes [5,6]. The synthetic convenience and environmental friendliness of this system aroused wide interests, and meanwhile, their high epoxidation activity and chiral induction in transformation of *cis*-, cyclic, and *tri*-substituted alkenes attracted more intentions for industrialization [7]. In addition, (salen)Mn(III) homogeneous catalysts (complexes) showed little credits in recycling that eventually hampered the road to industrial application [7]. On the basis of above progresses, it seemed highly significant to detect catalytic epoxidation activity of the combination of Ti(IV)/tartrate system with (salen)Mn(III) complex, which may integrate the benefits of two sources and bring about some encouraging results.

On the other hand, to address the environmental concerns and the economic considerations, modification of (salen)Mn(III) complexes to create heterogeneous catalysts was the key for recovery and reuse of catalyst. Therefore, many experiments leading to heterogenization of (salen)Mn(III) complexes were performed, including immobilization of (salen)Mn(III) monomers into Y zeolite [8], grafting of (salen)Mn(III) to polymers through covalent linkage [9], fixation of (salen)Mn(III) monomers onto porous solids through noncovalent linkages [10], as well as mixing (salen)Mn(III) into ionic liquids [11]. Overall, although various immobilizing strategies and many supporting materials had been applied during this process, the endeavors carried out so far have not been so successful, particularly regarding poor fresh catalysis or gradually declining recycling outputs. In practice, accessibility of substrate to metal center was usually limited by supporting materials [12], whose weak solubility in solvents led to leaching of active components from catalysts, which not only depressed recycling catalysis, but also contaminated products [12]. Therefore, it can be seen that developing new and effective supporting materials still deserves more endeavors.

In recent years, the employment of new supporting materials, particularly those bearing a highly ordered structure, appeared to be a really promising strategy to construct heterogeneous (salen)Mn(III) catalysts. Herein, the highly ordered structure may provide a large amount of uniform catalytic microenvironments for transformation along with high chemoselectivity and stereoselectivity [13]. Among these candidates, layered double hydroxide (LDH), for instance, a stable layered material facilitating an experimental composition of [M^2+^_1-*x*_M^3+^*_x_*(OH)_2_][A*^n^*^-^]*_x/n_*·*z*H_2_O, had aroused increasing attentions owing to their various applications in industry, including removal of chlorinated compounds from aqueous solution [14], adsorption and decontamination of radioactive elements [15], reduction of flammability [16], electrical applications [17], or catalysis [18]. Overall, owing to synthetic availability and low cost of LDH materials, applications of LDH would become more and more attractive in many fields.

In structural respects, the layers of LDH were bound together by van der Waals or electrostatic interactions, so interlayer spaces of LDH could be used through exfoliation or intercalation, which eventually made LDH a particularly attractive supporting material for creating various composites [19]. A sulphonato-(salen)Mn(III) was intercalated into Zn(II) and Mg(II)/Al(III) LDH by Anderson [20] and Choudary [21], in order to construct efficient epoxidation catalysts, indicating interlayer spaces of LDH were flexible enough to accommodate larger molecules. Furthermore, Wypych and co-workers also proved hydroxyl groups on LDH surfaces could be modified by (3-aminopropyl)triethoxysilane to support porphyrin complexes, further broadening applications of LDH [22]. At the same time, LDH materials showed obvious structural memory effects in pollutant adsorption applications [23], which may also improve catalyst endurance and play a key role in catalyst recycling.

**Figure 1:**
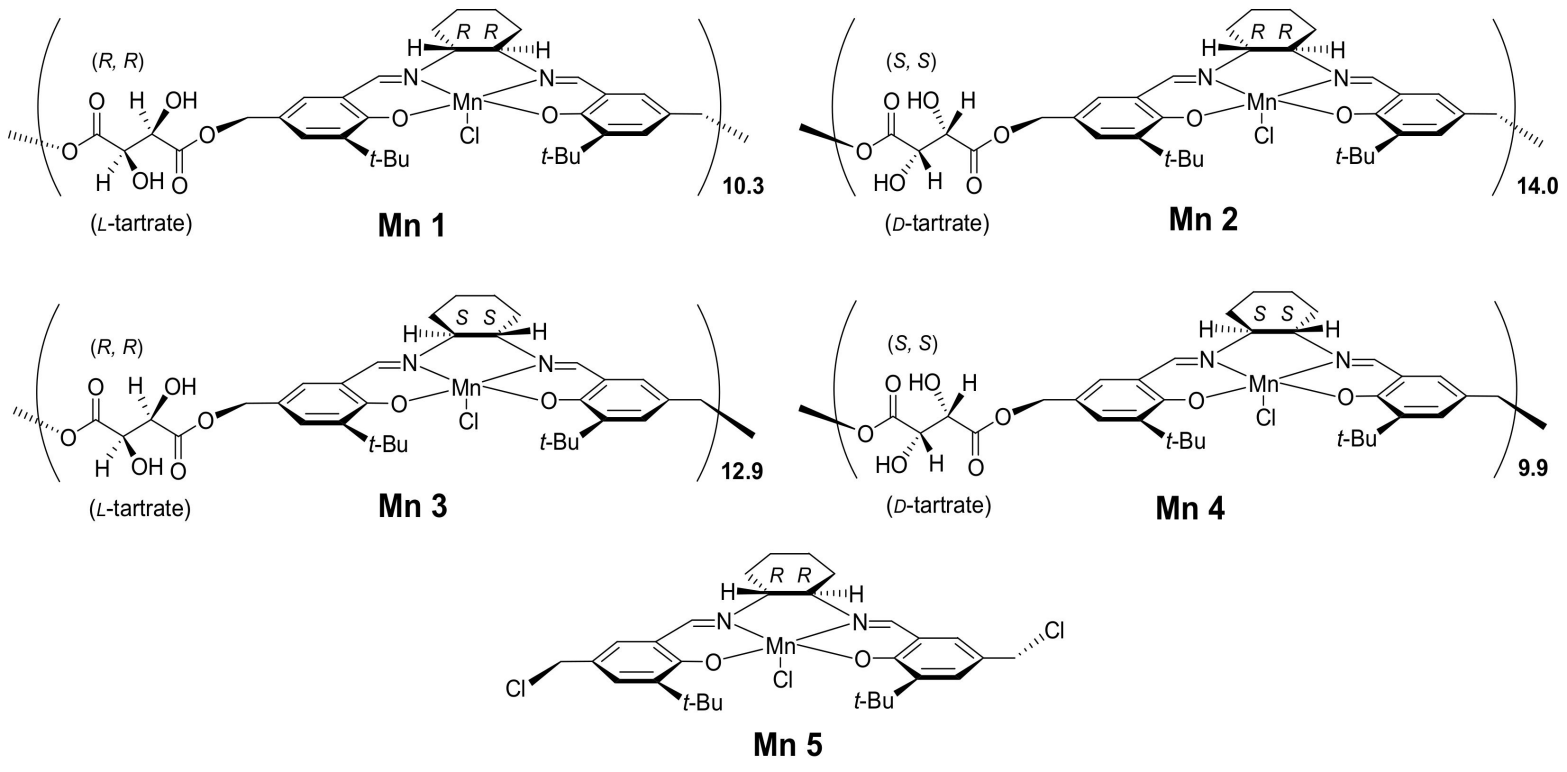
Structural illustration of the synthesized (tartrate-salen)Mn(III) polymers.

On the basis of the above progresses, four chiral (salen-tartrate)Mn(III) polymer complexes as well as one (salen)Mn(III) monomer complex were synthesized in this work and then applied into catalytic asymmetric epoxidation of alkenes (Figure 1). This part of the research was carried out in order to detect the possible synergistic effects of multiple chiral centers within one polymer complex over catalytic epoxidation reactions. Furthermore, a Zn(II)/Al(III) layered double hydroxide was prepared and then aminated for hosting (salen-tartrate)Mn(III) polymers through axial coordination, which were further used as catalysts for asymmetric epoxidation. In addition to the abovementioned advantages of LDH using as a catalyst, amination of LDH would give many coordination ligands for supporting (salen-tartrate)Mn(III) polymers, which not only seemed more rigid and endurable than other physically adsorbed immobilizations, but also fixed multiple chiral centers in order in support of accomplishing high chiral induction in catalysis. All in all, this work would contribute to heterogeneous asymmetric catalysis and corresponding materials science.

## 2. Experimental

### 2.1. Materials

The starting materials for synthesis of (tartrate-salen)Mn(III) polymer complexes including 1,2-diaminocyclohexane (mixture of isomers) (Sigma-Aldrich Inc., St. Louis, MO, USA); *L*-(+)- and *D*-(-)-tartaric acids (Sigma-Aldrich Inc., St. Louis, MO, USA); sodium *L*-(+)- and *D*-(-)-tartrate dihydrates (Sigma-Aldrich Inc., St. Louis, MO, USA); 2-*tert*-butylphenol(Sigma-Aldrich Inc., St. Louis, MO, USA); paraformaldehyde (Sigma-Aldrich Inc., St. Louis, MO, USA); tetrabutylammonium bromide (TBAB) (Sigma-Aldrich Inc., St. Louis, MO, USA); catalytic substrates such as styrene, α-methylstyrene (Sigma-Aldrich Inc., St. Louis, MO, USA), *trans*-stilbene (Sigma-Aldrich Inc., St. Louis, MO, USA), and indene(Sigma-Aldrich Inc., St. Louis, MO, USA); materials that were used for modification of supports like 3-aminopropyltrimethoxysilane (3-APTMS), Pluronic P123 (average *M*_n_, 5800), tetraethyl orthosilicate (TEOS) (Sigma-Aldrich Inc., St. Louis, MO, USA), and 3-chloroperoxybenzoic acid (*m*CPBA) (Sigma-Aldrich Inc., St. Louis, MO, USA), along with metal or non-metal salts; as well as HPLC-grade solvents were bought from Sigma-Aldrich Corporation without further purifications(Sigma-Aldrich Inc., St. Louis, MO, USA). The local suppliers provided organic solvents and silica gel of column and thin layer chromatography (all analytical reagents). At the same time, synthetic intermediates including (*R,R*)-1,2-diammoniumcyclohexane mono-(+)-tartrate salt [24], (*S,S*)-1,2-diammoniumcyclohexane mono-(-)-tartrate salt [24], 3-*tert*-butyl-5-chloromethyl-2-hydroxybenzaldehyde [24], Zn(II)/Al(III) LDH-[C_6_H_5_COO] (Zn/Al LDH, [Zn_2.09_Al_0.69_(OH)_5.23_]_1.00_[C_6_H_5_COO]_0.62_·H_2_O) [25], Mn5 [26], and iodosylbenzene (PhIO) [27] were synthesized according to literatures.

### 2.2. Characterization

H^1^ NMR of organic intermediates were tested on Bruker ADVANCE III (400MHz) (Bruker Corporation, Bruker ADVANCE III, Billerica, MA, USA). UV-Vis spectra were collected on Shimadzu UV-1800 (sample of 10^−3^ mol L^−1^ Mn in CH_2_Cl_2_, 290–550 nm) (Shimadzu Corporation, UV-1800, Kyoto, Japan). FT-IR spectroscopy was measured in KBr pellets over Bruker Tensor 27 spectrometer (400–4000 cm^−1^) (Bruker Corporation, Bruker Tensor 27 spectrometer, Billerica, MA, USA). The elemental analyses (C, H, and N) were carried out on Elementar VarioEL III. ESI-HRMS (positive molecular ions) were probed on microOTOF-Q II (Bruker.Daltonics production) (Bruker Corporation, microOTOF-Q II, Billerica, MA, USA). The number (*M*_n_)- and weight (*M*_w_)-average molecular and polydispersity indices (PDI, *M*_w_/*M*_n_) of four polymeric salen ligands were detected on gel permeation chromatography (GPC, Waters 1515–2414, Styragel HT3 THF column, 40 °C, THF as eluent, 1 mL min^−1^ flow rate, calibrated by polystyrene standard) (Waters Corporation, Waters 1515–2414, Milford, MA, USA). Metal contents of synthetic catalysts were detected by inductively coupled plasma atomic emission spectrometry (ICP-AES) on ICPE-9000 (Shimadzu Corporation, ICPE-9000, Kyoto, Japan). Optional rotations of chiral ligands were obtained on Perkin-Elmer 341 (λ = 587 nm, 25 °C, values formatted as absolute rotation ${\left[\alpha \right]}_{D}^{25}$, sample of 0.01 g mL^−1^ in CH_2_Cl_2_) (PerkinElmer, Inc., Perkin-Elmer 341, Waltham, MA, USA).

Porosity parameters including BET surface area, pore volume, pore radius, and pore size distribution of catalysts were determined on Micromeritics ASAP 2020 (testing N_2_ adsorption isotherms, 77.35 K) (Micromeritics Instrument Corporation, Micromeritics ASAP 2020, Norcross, GA, USA). Surface area was obtained by using multi-point Brunauer-Emmett-Teller (BET) method (*P*/*P*_0_ = 0.06–0.3). Total pore volume was calculated from adsorbed N_2_ (*P*/*P*_0_ = 0.97); pore volume and pore radius were determined by using Barrett-Joyner-Halenda (BJH) method. Bulk densities of the synthesized catalysts were detected on SOTAX TD2 density detector (CAMAG Corporation, SOTAX TD2, Muttenz, Switzerland). X-ray photoelectron spectroscopy (XPS) were carried out on Kratos Axis Ultra DLD (Kratos Analytical Ltd., Kratos Axis Ultra DLD, Manchester, UK), irradiation source was monochromatic Al Kα X-ray (1486.6 eV). The X-ray diffraction (XRD) of powdered catalysts was tested on Shimadzu XRD-6000 (Cu-Ka_1_, λ = 1.54059 Å) (Shimadzu Corporation, XRD-6000, Kyoto, Japan), and diffractions were collected (2*θ* = 4–55°, 0.02° intervals). Scanning electron microscopy (SEM) was carried out on JSM-6700F (JEOL) (JEOL, Ltd., JSM-6700F, Tokyo, Japan). Particle size and zeta potential of the synthesized catalysts were measured on Zetasizer Nano ZS90, Malvern (in CH_2_Cl_2_, 298 K) (Malvern Panalytical Ltd., Zetasizer Nano ZS90, Malvern, UK).

Thin layer chromatography (TLC) was performed on glass plates coated with GF_254_ silica gel (coloration in phosphomolybdic acid (PMA)/ethanol, 5 wt.%). Conversions of substrates and enantiomeric excesses of epoxide products were both detected by chiral HPLC (Daicel Chiralcel OD-H column, 150 mm × 4.6 mm; 5 μm particle size; mobile phase: *n*-hexane/2-propanol, 97/3, *v*/*v*; flow rate: 1.0 mL min^−1^; column temperature: 300 K; pressure: 5.0–7.0 MPa; sample concentration: 1.0 mg mL^−1^ in *n*-hexane; 10 μL injected; Waters chromatograph, system controller: Waters 1525, binary hplc pump; UV-Vis detector: Waters 2998, photodiode array detector; UV detection: 242 nm, λ = 210–400 nm).

### 2.3. Synthesis of Chiral Dimeric Salicylaldehyde (Compound 3, Scheme 1)

**Scheme 1:**
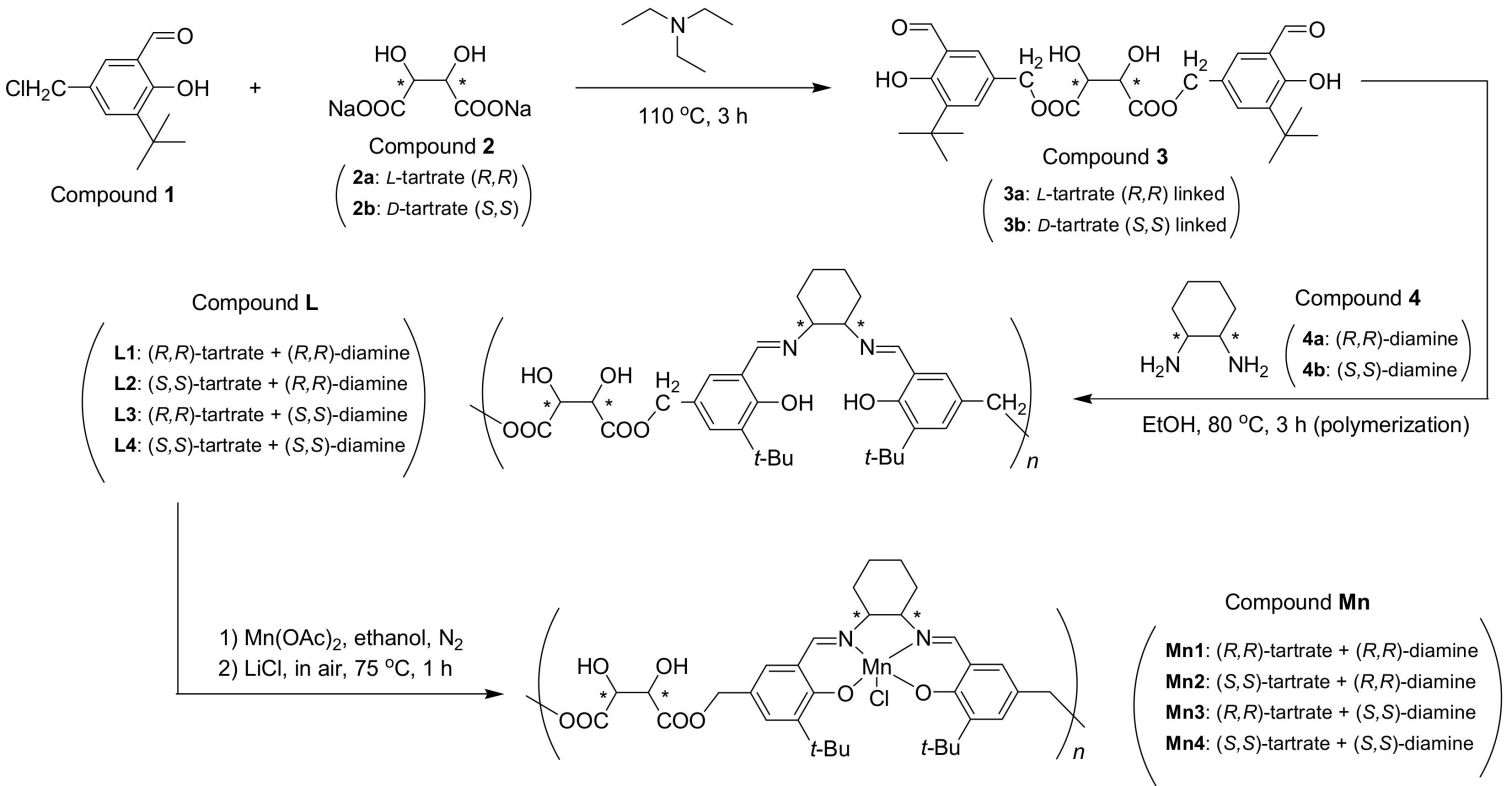
Synthesis of (tartrate-salen)Mn(III) polymer catalysts.

As shown in Scheme 1, 3-*tert*-butyl-5-chloromethyl-2-hydroxybenzaldehyde (Compound 1, Scheme 1; 3.25 g, 14.4 mmol), sodium *L*-(+)-tartrate dihydrate (compound 2a, Scheme 1; 1.65 g, 7.2 mmol), and dry triethylamine (30 mL) were mixed into a round-bottomed flask (250 mL). With continuous stirring, the resulting orange solution was heated at 110 °C for 3 h. It can be seen that small crystals (NaCl) were gradually precipitated from the purple solution when the reaction was continued. Then, the solvent was removed under reduced pressure, after the total solution was cooled to room temperature. The CH_2_Cl_2_ (100 mL) was subsequently added to dilute residue. The CH_2_Cl_2_ layer obtained was washed with water (3 × 50 mL) and brine (3 × 50 mL), then dried over anhydrous Na_2_SO_4_, and filtered. After the removal of solvent by using rotary evaporation, residue was further purified by using column chromatography (SiO_2_, 200–300 mesh; petroleum ether/ethyl acetate, 6/1, *v*/*v*, adding a few drops of Et_3_N) to give chiral salicylaldehyde dimer (compound 3a, Scheme 1; yellow sticky solid, 1.49 g, 39% yield). ^1^H NMR (CDCl_3_, 400 MHz) δ_H_ (ppm): 1.27 (9H, s), 3.95 (4H, s), 4.55 (2H, s), 7.18–7.23 (2H, m), 7.50–7.61 (2H, m), 9.85 (2H, s). FT-IR (tested in KBr pellets) σ (cm^−1^): 3432 (m), 3398–3280 (br, s), 2939 (s), 2856 (s), 1719 (vs). ESI-HRMS (positive, m/z): 553.6002 (Calcd. for [M+Na]^+^ 553.5519). ${\left[\alpha \right]}_{D}^{25}$ = −65 (*c* = 0.01 g mL^−1^, CH_2_Cl_2_). Ideal formula of 3a is C_28_H_34_O_10_. Anal. Calcd.: C, 63.4; H, 6.4. Found: C, 62.8; H, 7.0. Synthesis of 3b was identical to 3a except for replacement of sodium *L*-(+)-tartrate dihydrate with sodium *D*-(-)-tartrate dihydrate (Section S1, Supplementary data).

### 2.4. Synthesis of Chiral Ligands (Compound L, Scheme 1)

(*R,R*)-1,2-diammoniumcyclohexane mono-(+)-tartrate salt (0.45 g, 1.7 mmol), anhydrous K_2_CO_3_ (0.47 g, 3.4 mmol), and distilled water (15 mL) were mixed into a round-bottomed flask (250 mL) at room temperature. Then, dry ethanol (6 mL) was introduced under magnetic stirring. The resulting cloudy solution was heated at 75 °C for 2 h under stirring, and then cooled to room temperature. Next, this solution was extracted by CH_2_Cl_2_ (4 × 5 mL) to obtain free diamine (Compound 4a, Scheme 1). The CH_2_Cl_2_ phase was then slowly added to a pre-prepared ethanol solution of compound 3a (0.9 g, 1.7 mmol, in 20 mL, Scheme 1) at room temperature under stirring. The orange solution was refluxed at 80 °C for 3 h, whose solvent was evaporated under reduced pressure. The crude product thus obtained was dissolved in CH_2_Cl_2_ (30 mL), the organic layer was washed with distilled water (50 mL), brine (50 mL), dried over anhydrous Na_2_SO_4_, and then filtered. Solvent was removed by using rotary evaporation, and Compound L1 was obtained as yellow sticky solid (0.96 g). ^1^H NMR (400 MHz, CDCl_3_) δ_H_ (ppm): 1.41 (9H, s), 1.40–1.56 (8H, m), 2.33 (2H, s), 3.93 (4H, s), 4.53 (2H, s), 7.40–7.44 (2H, m), 7.50–7.55 (2H, m), 9.88 (2H, s). FT-IR (KBr) σ (cm^−1^): 3442 (br, s), 2958 and 2867 (m), 2930 (m), 1772 (w), 1649 (s), 1559 (w), 1267 (w). ${\left[\alpha \right]}_{D}^{25}$ = −116 (*c* = 0.01 g mL^−1^, CH_2_Cl_2_). *M*_n_ = 6262, *M*_w_ = 13150, PDI (*M*_w_/*M*_n_) = 2.1. Based on *M*_n_, number of tartrate-salen monomers was 10.3, then the ideal formula of L1 was deduced as (C_4_H_4_O_6_·C_30_H_40_O_2_N_2_)_10.3_. Anal. Calcd.: C, 67.1; H, 7.2; N, 4.6. Found: C, 66.8; H, 6.7; N, 5.5. Synthesis of Compounds L2, L3, and L4 were identical to L1 except for substitution of chiral tartrate and diamine counterparts (Section S2, Supplementary data).

### 2.5. Synthesis of (Tartrate-Salen)Mn(III) Polymer Complexes (Compound Mn, Scheme 1)

Compound L1 (Scheme 1, 0.81 g), Mn(OAc)_2_·4H_2_O (0.62 g, 2.55 mmol), and anhydrous ethanol (10 mL) were mixed into a round-bottomed flask (100 mL), which was then refluxed at 75 °C for 3 h under N_2_ protection. After protection was removed, LiCl·H_2_O (0.46 g, 7.65 mmol) was added. The solution that obtained was further stirred at 75 °C for 1 h in air, then solvent was evaporated under reduced pressure, and the resulting brown powders were collected and thoroughly washed with distilled water (3 × 30 mL), then Compound Mn1 was obtained as brown powders (0.76 g). FT-IR (in KBr pellets) σ (cm^−1^): 3433 (br, s), 2957 and 2868 (m), 2930 (m), 1741 (w), 1618 (s), 1544 (w), 1268 (w), 567 (w). ${\left[\alpha \right]}_{D}^{25}$ = +129° (*c* = 0.01 g mL^−1^, CH_2_Cl_2_). According to L1, the ideal formula of Compound Mn1 was summarized as (C_4_H_4_O_6_·C_30_H_38_O_2_N_2_MnCl·2H_2_O)_10.3_. Anal. Calcd.: C, 55.7; H, 6.2; N, 3.8. Found: C, 56.3; H, 6.1; N, 4.5. Mn^3+^ content was 1.10 mmol g^−1^ by ICP-AES. The syntheses of other polymer complexes (Compounds Mn2, Mn3, Mn4) were identical to Mn1 (Section S3, Supplementary data).

### 2.6. Synthesis of Free Amino-Functionalized Zn(II)/Al(III) LDH-[C_6_H_5_COO] (amino-LDH, Scheme 2)

**Scheme 2:**
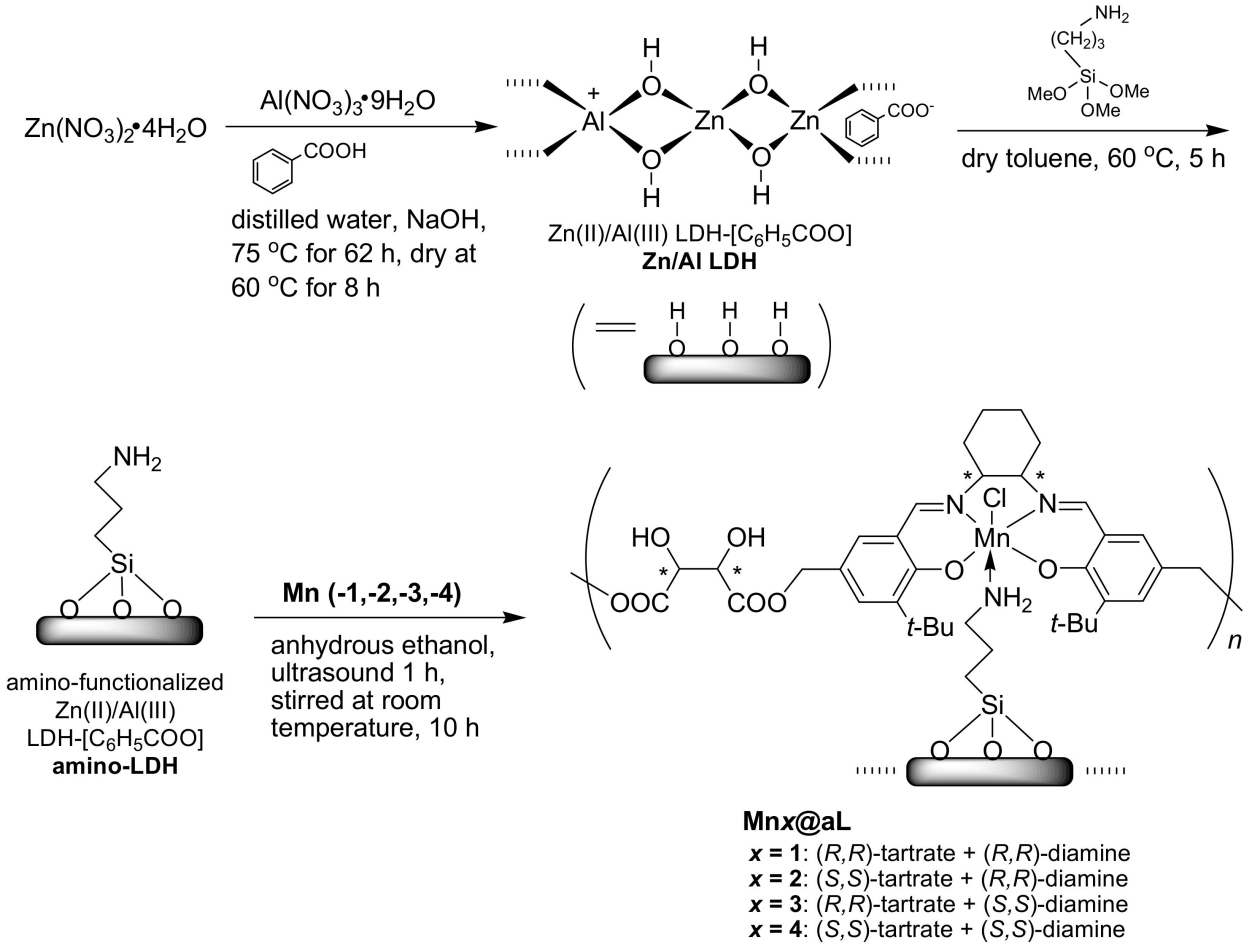
Immobilization of (tartrate-salen)Mn(III) polymers onto aminated LDH.

As shown in Scheme 2, Zn/Al LDH (1.0 g) was dispersed into dry toluene (150 mL) by using ultrasound for 2 h, then 3-APTMS (15.0 g, 83.6 mmol) was introduced at room temperature. The suspension was further heated at 60 °C for 5 h under vigorous stirring, then filtered under reduced pressure. Crude product was carefully washed by dry toluene (3 × 50 mL), then amino-LDH was obtained as white solids (0.96 g). FT-IR (KBr) σ (cm^−1^): 3747 and 3673 (both w), 3649 and 3540 (both w), 3444 (m), 2936 (w), 1699 (m), 1599 (m), 1194 (w). Ideal formula of amino-LDH was [Zn_2.09_Al_0.69_(OH)_5.23_]_1.00_[C_6_H_5_COO]_0.62_[C_3_H_8_NO_3_Si]_0.21_. Anal. Calcd.: C, 17.2; H, 2.8; N, 0.8. Found: C, 17.5; H, 3.1; N, 0.8.

### 2.7. Synthesis of Amino-LDH-Supported (Tartrate-Salen)Mn(III) Polymers (Mnx@aL, x = 1, 2, 3, 4)

Compound Mn1 (Scheme 1, 0.20 g), amino-LDH (0.40 g), and anhydrous ethanol (20 mL) were mixed into a round-bottomed flask (250 mL), which was then dispersed under ultrasound for 1 h, and stirred for 10 h at room temperature. After total solvent was removed by rotary-evaporation, the residue was collected and washed with anhydrous ethanol (2 × 5 mL). Mn1@aL was obtained as yellowish-brown powders (0.42 g). FT-IR (KBr) σ (cm^−1^): 3711 and 3674 (both w), 3648 and 3589 (both m), 3443 (br, s), 2960 and 2869 (both w), 2925 (w), 1770 (w), 1697 (m), 1626 (m), 1558 (m), 1261 (w), 1134 (s), 876 (w), 566 (w), and 420 (w). The ideal formula of Mn1@aL was [Zn_2.09_Al_0.69_(OH)_5.23_]_1.00_[C_6_H_5_COO]_0.62_[C_3_H_8_NO_3_Si]_0.21_[C_4_H_4_O_6_·C_30_H_38_O_2_N_2_MnCl]_0.06_. Anal. Calcd.: C, 21.6; H, 3.2; N, 1.1. Found: C, 20.3; H, 2.6; N, 1.1. Mn^3+^ content was 0.18 mmol g^−1^ determined by ICP-AES. Mn*x*@aL (*x* = 2, 3, 4) were prepared according to the same process (Section S4, Supplementary data).

### 2.8. Catalytic Activity

Alkene substrate (1 mmol), catalyst (1.5–6 mol% Mn according to alkene), oxidant (PhIO or *m*-CPBA, 1.2 mmol), NH_4_OAc (co-catalyst, 0.12 mmol), and CH_2_Cl_2_ (or other solvents, 5 mL) were mixed into a round-bottomed flask (100 mL) under ice bath (0 °C). The mixture was monitored by TLC with PMA coloration under vigorous stirring (petroleum ether/CH_2_Cl_2_, 2/1, *v*/*v*; *R*_f_ of styrene, α-methylstyrene, *trans*-stilbene, and indene: 0.89, 0.86, 0.72, 0.75; *R*_f_ of corresponding epoxides: 0.26, 0.23, 0.36, 0.32). After 6 h, the mixture was concentrated to dryness under reduced pressure, residue was extracted by using *n*-hexane (3 × 5 mL), and the left solid catalysts were combined with consumables for recycling. Hexane layer was concentrated under reduced pressure, and crude product was purified by a short column chromatography (alkaline Al_2_O_3_; petroleum ether/CH_2_Cl_2_, 2/1, *v*/*v*, a few drops of Et_3_N), then both conversion and e.e. value were reported on chiral HPLC.

## 3. Results and Discussion

### 3.1. Design and Synthesis of Catalysts

Design of the present catalysts was a rational combination of different chiral sources for one conversion target. Once *L*- and *D*-tartrate were combined with (*R,R*)- or (*S,S*)-1,2-diammoniumcyclohexane, four resulting polymer complex catalysts (Mn*x*, *x* = 1, 2, 3, 4) should have short-range ordering. Herein, the monomer Mn5, however, appeared as a comparative criteria to illustrate functions of tartrate linkers (Figure 1). Taking into account flexibility of the present four polymer complexes, they were subsequently immobilized over Zn/Al LDH.

The Mn*x* (*x* = 1, 2, 3, 4) were synthesized according to the route that is shown in Scheme 1. At first, the connection of 3-*tert*-butyl-5-chloromethyl-2-hydroxybenzaldehyde (Compound 1, Scheme 1) with sodium tartrate dihydrate (Compound 2, Scheme 1) was realized along with acceptable yields, when triethylamine were selected as base and solvent (Scheme 1). Sensitivity of this kind of transformation (esterification) was determined by the untouched tartrate, and other strong bases including LiOH, NaOH, or K_2_CO_3_ would destroy tartrate and were not available in practice. In the meantime, free chiral diamine should be extracted from alkaline solution due to the same reason, before its polymerization with bis-aldehyde (Scheme 1).

Furthermore, due to low cost and synthetic availability, Zn/Al LDH was prepared according to the method shown in Scheme 2. In order to build more linkers on support, Zn/Al LDH was aminated, leaving many amino groups for accommodating (tartrate-salen)Mn(III) polymers, which actually fixed the flexible Mn*x* (*x* = 1–4) into a confined environment (Scheme 2).

### 3.2. FT-IR Spectroscopy

**Figure 2:**
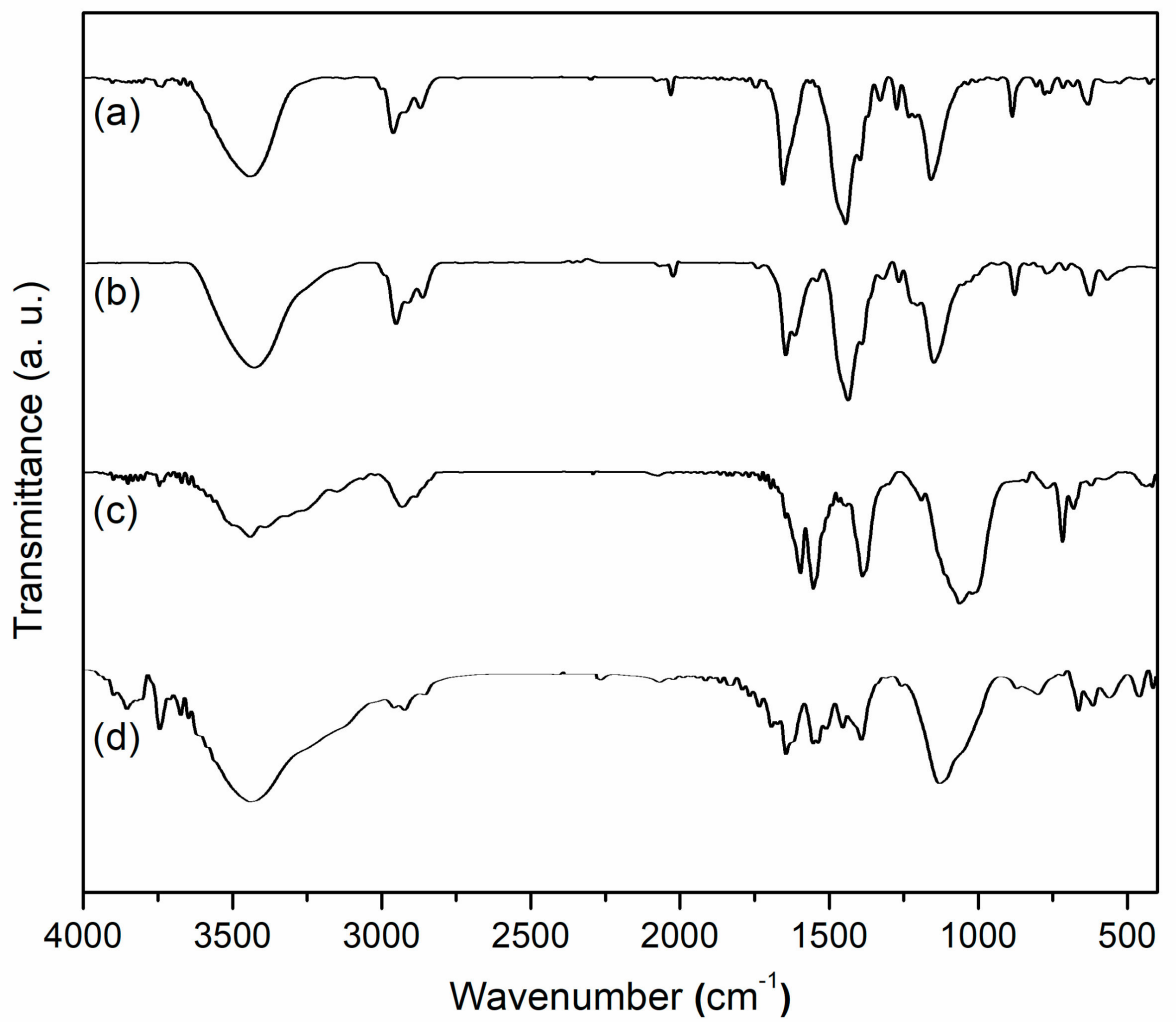
FT-IR spectra of (**a**) L1, (**b**) Mn1, (**c**) amino-LDH, (**d**) Mn1@aL.

FT-IR spectroscopy of the synthesized samples including L1, Mn1, amino-LDH, and Mn1@aL are consecutively shown in Figure 2. The L1 showed vibration bands at 3442 (ArO–H, O–H on tartrate, overlapped), 2958 and 2867 (C-H on methyl), 2930 (C-H on methylene), 1772 (tartrate), 1649 (C=N), 1559 (C–O), and 1267 (Ar–OH) cm^−1^ (Figure 2a). The Mn1 provided corresponding vibrations at 3433, 2957 and 2868, 2930, 1741, 1618, 1544, and 1268 (Ar–OMn) cm^−1^, along with a new vibration of Mn-O stretching at 567 cm^−1^ (Figure 2b). Based on the comparison of Mn1 with L1, the red shift was observed on C=N stretching from 1649 (L1) to 1618 cm^−1^ (Mn1) (Figure 2a,b), which could be assigned to the coordination of nitrogen to Mn^3+^ that reduced electron density on C=N bond [28]. On the other hand, red shift of tartrate from 1772 (L1) to 1741 cm^−1^ (Mn1) and that of C-O on tartrate from 1559 (L1) to 1544 cm^−1^ (Mn1) could be attributed to coordination of tartrate part to Mn^3+^ (Figure 2a,b), probably leading to a curved configuration of Mn1 more than a linear one.

FT-IR of Mn1@aL (Figure 2d) showed vibration bands at 3711 and 3674 (AlO–H), 3648 and 3589 (ZnO–H), 3443 (O–H on tartrate or N–H, overlapped), 2960 and 2869 (C–H on methyl), 2925 (C–H on methylene), 1770 (tartrate), 1697 (C=O on benzoate), 1626 (C=N), 1558 (C-O on tartrate), 1261 (Ar–O), 1134 (Si-C), 876 (Si-O), 566 (Mn–O), and 420 (Mn-N) cm^−1^, being recognized by comparison with data of amino-LDH (Figure 2c) covering 3747 and 3673 (AlO–H), 3649 and 3540 (ZnO–H), 3444 (N–H), 2936 (C–H on methylene), 1699 (C=O on benzoate), 1599 (N–H), and 1194 (Si–C) cm^−1^. Probably because axial coordination of nitrogen with Mn^3+^ increased electron density of manganese center and imine bond, a blue shift of C=N from 1618 (Mn1) to 1626 (Mn1@aL) cm^−1^ was observed (Figure 2c,d) [29]. This axial coordination also played an important role in configuration control of the polymer complex. Blue shift of tartrate ester from 1741 to 1770 cm^−1^ and that of C–O on tartrate from 1544 to 1558 cm^−1^ were both obtained from Mn1 to Mn1@aL (Figure 2b,d), revealing degradation of coordination between the tartrate and manganese center, which also indicated Mn1 were flattened at the LDH surface more than being rolled.

### 3.3. UV-Vis Spectroscopy

**Figure 3:**
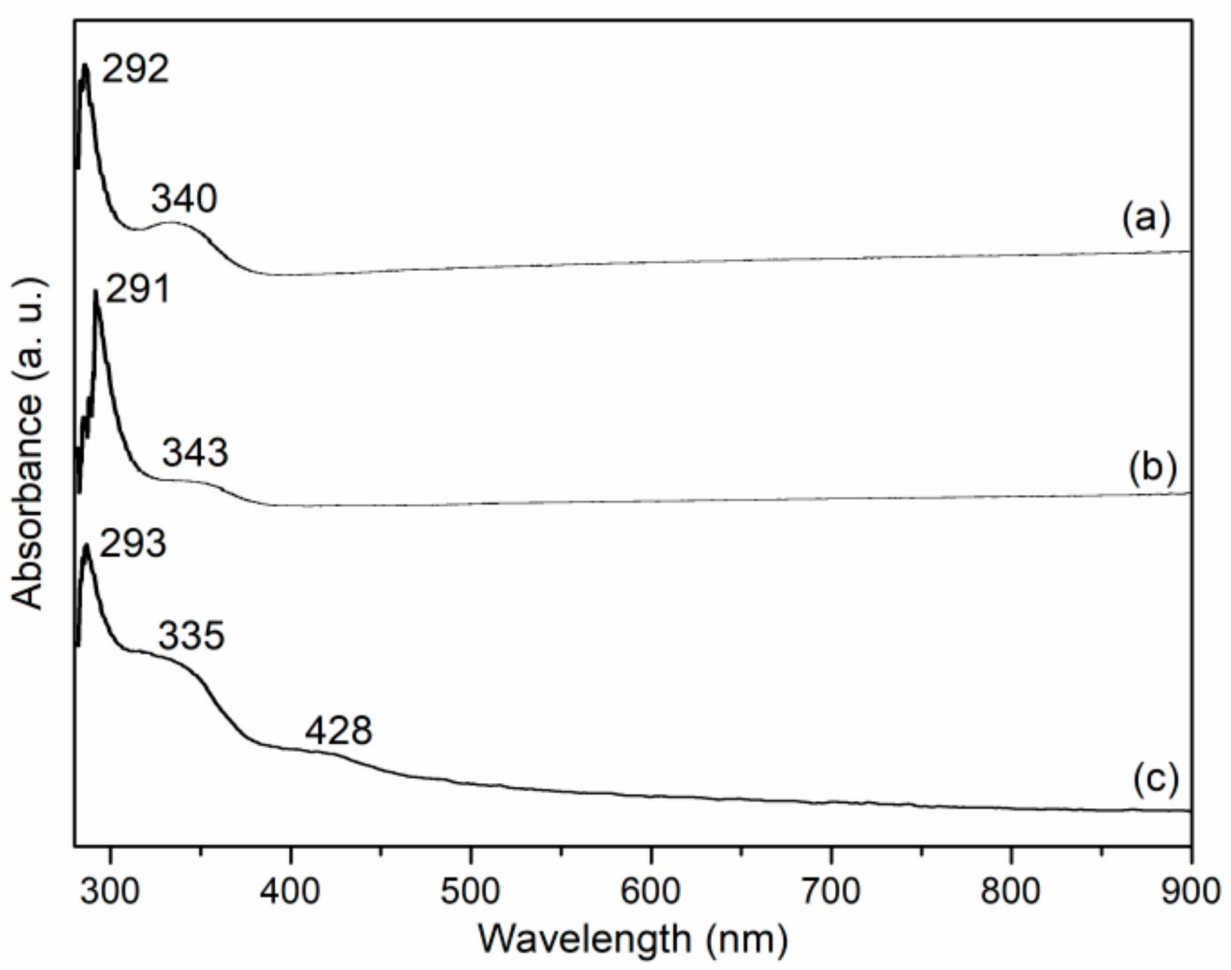
UV-Vis spectra of (**a**) L1, (**b**) Mn1, (**c**) Mn1@aL.

UV-Vis spectra of L1, Mn1 and Mn1@aL are shown in Figure 3. For L1, the bands that appeared at 292 and 340 nm could both be ascribed to charge transfer transitions of salen ligands (Figure 3a), while Mn1 showed corresponding responses at 291 and 343 nm (Figure 3b), exhibiting two small shifts, probably because of the coordination of manganese [26]. If Mn1 was axially immobilized over amino-LDH, charge transfer transition of salen ligand was shifted to 293 and 335 nm, together with a new ligand-to-metal charge transfer transition that occurred at 428 nm (Figure 3c). Herein, two shifts (Figure 3b,c) indicated the interaction between Mn1 and amino-LDH, confirming immobilization was effective [26].

### 3.4. Nitrogen Physisorption

**Figure 4:**
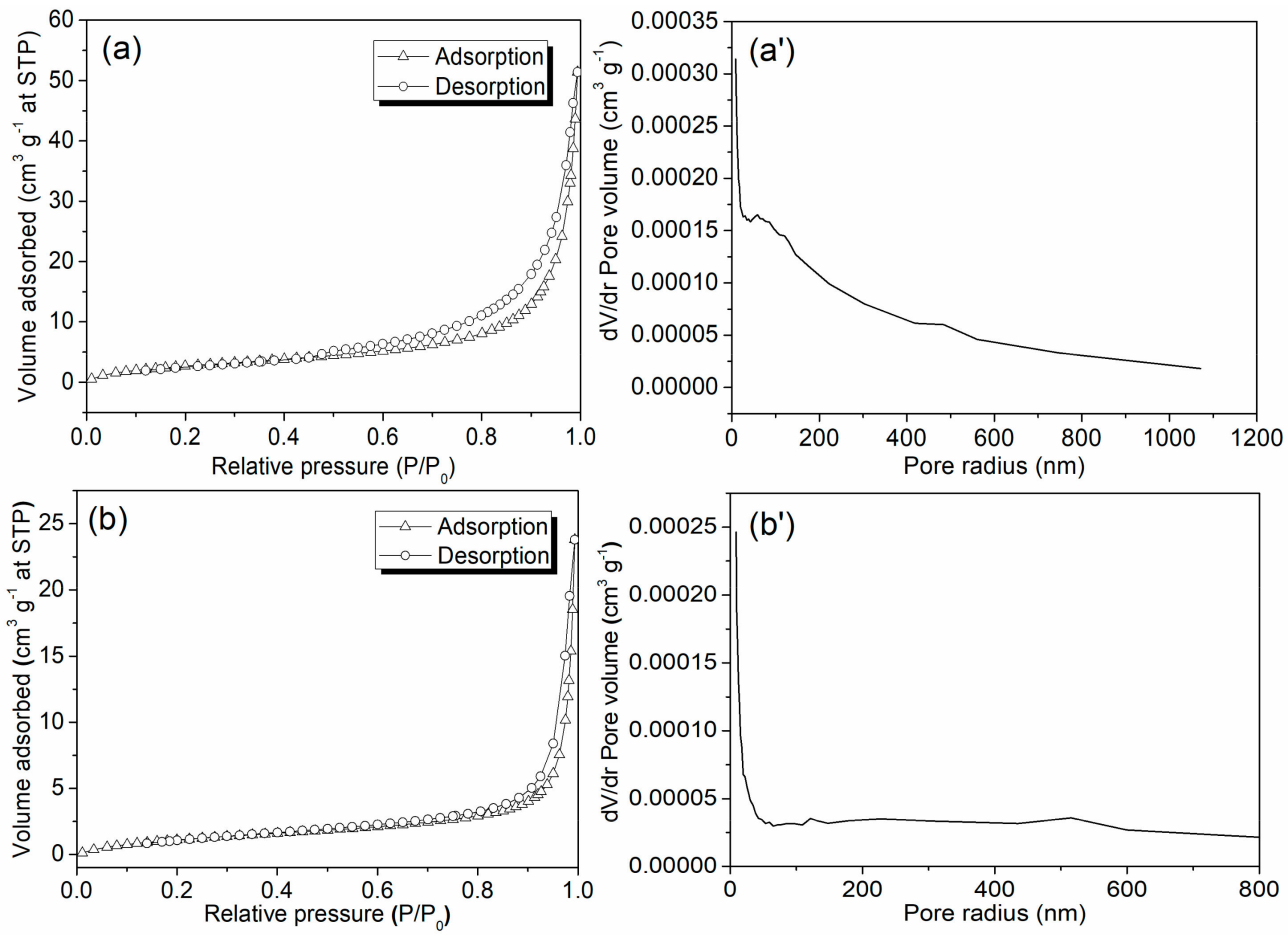
N_2_ adsorption-desorption isotherms and pore size distributions of Zn/Al LDH (**a**,**a’**) and Mn1@aL (**b**,**b’**).

Both Zn/Al LDH and Mn1@aL showed a type II isotherm, but Zn/Al LDH had a H3 hysteresis loop, while Mn1@aL did not show an obvious hysteresis loop (Figure 4) [30]. Therefore, Zn/Al LDH should have a layered structure that represented unrestricted monolayer-multilayer adsorption of nitrogen [30]. Difference on hysteresis loop revealed that Zn/Al LDH were probably an aggregate of plates with slit-shaped pores, but Mn1@aL looked like a non-porous material, corresponding to its flat pore size distribution (Figure 4b’). Back to the way Mn1@aL formed, 3-APTMS supposedly filled in the tiny pores on every plate of Zn/Al LDH through self-assembling [22], and its further coordination with Mn1 led to this result. Naturally, Mn1@aL had a larger pore radius but smaller pore volume and BET surface area than Zn/Al LDH (Table 1).

### 3.5. Powdered XRD

**Figure 5:**
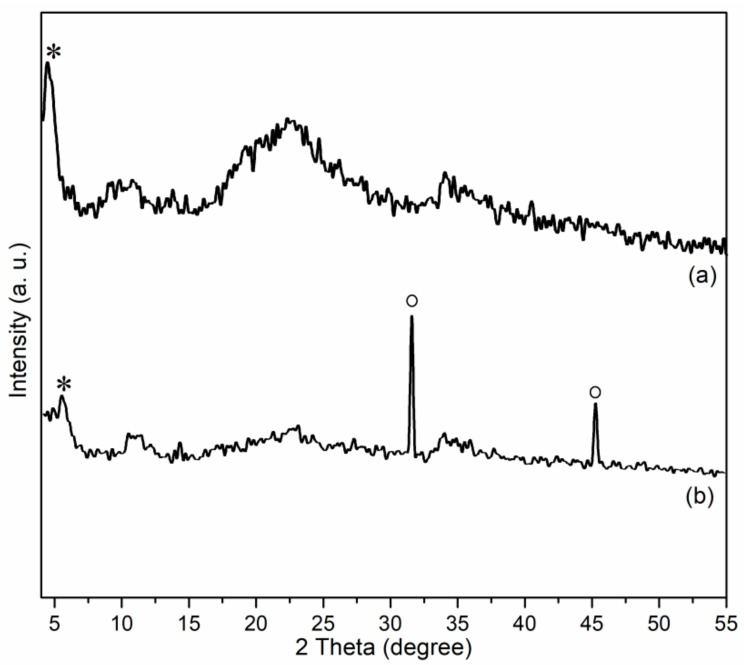
Powdered XRD patterns of (**a**) Zn/Al LDH and (**b**) Mn1@aL.

Figure 5 showed powdered XRD spectra of Zn/Al LDH and Mn1@aL. First of all, the present Zn/Al LDH showed a basal spacing of 20.24 Å (2*θ* = 4.36°, asterisk, Figure 5a), which is smaller than that of 26.2 Å of Mg-Al LDH [22], but much larger than that of 7.54 Å of Zn-Al LDH (CO_3_^2−^ as anion) [23], indicating both cation and anion affected basal spacing of LDH materials. Furthermore, Zn/Al LDH also showed several broad and flat bands at 2*θ* = 7.5–55.0° (Figure 5a), which are different with those found in Zn-Al LDH (CO_3_^2−^ as anion) [23], mainly because benzoate anion has a much poorer crystallinity than carbonate.

After immobilization, the sample obtained (Mn1@aL) showed a smaller basal spacing (16.41 Å, 2*θ* = 5.36°, asterisk in Figure 5b) than the support (Zn/Al LDH of this work, 20.24 Å), suggesting that many guest molecules were dispersed into layers of LDH through immobilization (Scheme 2). Mn1@aL also showed two sharp diffraction peaks (circles in Figure 5b), which could be ascribed to 200 (2*θ* = 31.53°) and 220 (2*θ* = 45.19°) diffractions of aluminum(II) oxide (AlO) phase (PDF No. 75–0278), probably stemming from the reduction of Al^3+^ of Zn–Al–[C_6_H_5_COO] LDH with 3-APTMS during immobilization (Scheme 2). Furthermore, in other regions, the diffraction peaks became wide and flat (Figure 5b), similar to the reported tendency [22,23,31], indicating the poor crystallinity of synthetic sample after immobilization with 3-APTMS (Scheme 2), and the effective immobilization process as well (Scheme 2).

### 3.6. Chemical Composition

Elemental analyses, metal contents, and molar ratio of N to Mn were summarized in Table 2, affording a macroscopic evaluation on chemical composition of synthetic samples. Based on the formula of Zn/Al LDH ([Zn_2.09_Al_0.69_(OH)_5.23_]_1.00_[C_6_H_5_COO]_0.62_·H_2_O), elemental analysis and ICP-AES, amino-LDH was deduced as [Zn_2.09_Al_0.69_(OH)_5.23_]_1.00_[C_6_H_5_COO]_0.62_[C_3_H_8_NO_3_Si]_0.21_, where presence of C_6_H_5_COO^−^ was witnessed by FT-IR adsorption at 1699 cm^−1^ (Figure 2c). Mn1@aL was further obtained as [Zn_2.09_Al_0.69_(OH)_5.23_]_1.00_[C_6_H_5_COO]_0.62_[C_3_H_8_NO_3_Si]_0.21_[C_4_H_4_O_6_·C_30_H_38_O_2_N_2_MnCl]_0.06_, in the light of characteristic IR adsorptions belonging to its functional groups (Figure 2d). However, the higher molar ratio of N/Mn verified that amino groups were excessive so as to saturate all manganese centers.

### 3.7. X-ray Photoelectron Spectroscopy

Binding energy and surface atomic composition of synthetic products were summarized in Table 3. In general, molar ratios of Al to Zn on surfaces of Zn/Al LDH and amino-LDH were both 2.0 (Table 3), but corresponding average values were fixed at 0.33 (Table 2), revealing that Al^3+^ were focused on exterior surface, and Zn^2+^ on interiority of LDH layers. Traces of nitrogen on the Zn-Al LDH surface mainly came from starting materials [25], but a higher molar ratio of nitrogen on amino-LDH was derived from coupling of 3-APTMS. Probably due to attachment of Mn1, carbon composition of Mn1@aL increased, but those of Si, N, and Zn declined sharply (Table 3), which suggested amino-LDH were tightly sealed by Mn1. Furthermore, atomic percentage of Mn was 0.4% on the surface of Mn1@aL, but average Mn of Mn1@aL was 0.2% based on its ideal formula, revealing interlayer spaces of Mn1@aL were filled with Mn1, but its content was sparser that that on exterior surfaces.

### 3.8. Scanning Electron Microscopy

**Figure 6:**
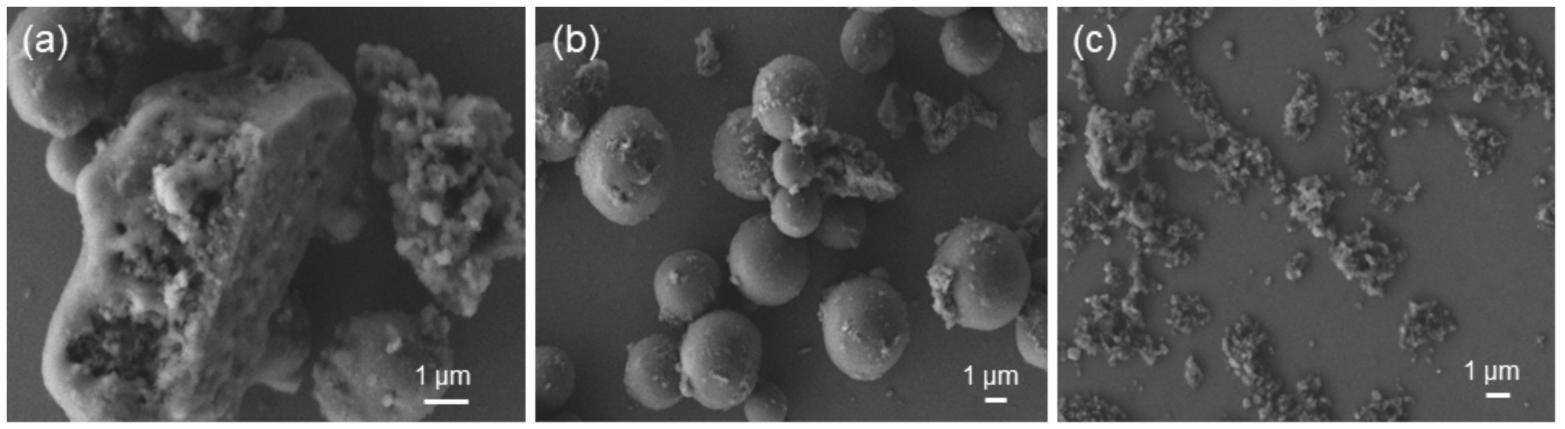
SEM images of (**a**) Zn/Al LDH, (**b**) amino-LDH, (**c**) Mn1@aL.

Size and morphology of Zn/Al LDH, amino-LDH, and Mn1@aL, were characterized by scanning electron microscopy, as shown in Figure 6. A planar surface could be found in Zn/Al LDH (Figure 6a), but unexpectedly its attachment to 3-APTMS created spheres with diameters ranging from 3 μm to 6 μm (Figure 6b). During immobilization of Mn1, the morphology of the synthetic product changed again, spherical structure collapsed and Mn1@aL was obtained as much smaller particles (Figure 6c).

### 3.9. Study of Catalytic Conditions

First of all, the synthesized catalysts (from Mn1 to Mn4) were quite soluble in many organic solvents including dichloromethane, acetone, diethyl ether, and acetonitrile, while they were less soluble in *n*-hexane and ethanol, but showed very poor hydrophilicity. Thus, to optimize catalytic conditions, enantioselective epoxidation of styrene was carried out in these solvents at 0 °C by using Mn1 as a catalyst, in which iodobenzene (PhIO) was employed as an oxidant. According to catalytic results that are shown in Table 4, it can be seen that catalytic conversion, enantioselectivity, and turnover frequency were highly affected by the solvent. In practice, Mn1 was almost insoluble in water, which was aggregated and deposited on the wall or bottom of the flask during the reaction, so interaction between Mn1 and substrate at the molecular level seemed to be very poor, and conversion and enantioselectivity were depressed simultaneously (entry 6, Table 4). Another protonic solvent, ethanol, appeared to be inappropriate also, although a slightly improved result was obtained compared to water (entries 5 vs. 6). More promising enantioselectivity and acceptable conversion were derived from the use of non-protonic solvents such as dichloromethane or *n*-hexane (entries 7 and 4), probably because the non-protonic solvents could promote S_N_2 reactions like epoxidation more than S_N_1 [32]. Catalyst loading also played a key role in outputs such as conversion and enantioselectivity. Although the highest conversion (26%) was realized by using the largest catalyst loading (6 mol%) among three dichloromethane facilitated reactions (entries 9 vs. 7 and 8), its corresponding e.e. was lower than that found in 3 mol% (entries 7 vs. 9). On the basis of the above experiments, dichloromethane and 3 mol% of catalyst loading were optimized for the following reactions.

### 3.10. Homogeneous Catalysis

The synthesized polymer catalysts (Mn1 to Mn4) were introduced to asymmetric epoxidation of unfunctionalized alkenes such as styrene, α-methylstyrene, *trans*-stilbene, and indene. The monomer, Mn5, was also selected as comparative criteria in order to observe the roles of tartrate spacers in catalyst activity or chiral induction. As shown in Table 5, all reactions proceeded well and would be accomplished within 6 h under monitoring of TLC (PMA coloration). Furthermore, iodobenzene (PhIO) was a unique oxidant that was almost insoluble in dichloromethane, thus the oxygen transfer occurred slowly on PhIO surface, naturally leading to comparatively higher e.e. values, along with less abundant conversions. This tendency was quite different with those found in *m*CPBA oxidized epoxidations (entries 2 vs. 1, 8 vs. 7, 14 vs. 13, and 20 vs. 19, Table 5).

The Mn1, derived from the linkage of (*R,R*)-salen with (*R,R*)-tartrate, provided the best enantioselectivity as well as acceptable conversion in asymmetric epoxidation of styrene (entry 1, Table 5), but its enantiomer Mn4, the combination of (*S,S*)-salen with (*S,S*)-tartrate, could not show equal results (entries 5 vs. 1, Table 5). Furthermore, Mn1 showed much better e.e. than Mn2, suggesting enantioselectivity would be improved when both tartrate linker and salen had the same (*R,R*)-configuration (entries 1 vs. 3, Table 5). In addition, chiral induction of Mn1 looked more prospective than Mn5 too (entries 1 vs. 6, Table 5), further indicating the conjunction of (*R,R*)-tartrate with (*R,R*)-salen was advantageous in epoxidation of styrene. In association with the red shift of tartrate that was found in FT-IR spectra of L1 and Mn1 (curves a and b, Figure 2), it can be seen that chiral tartrate should affect chiral induction through its coordination with Mn center. Nevertheless, configuration of major styrene oxides was still determined by chirality of salen (entries 1, 2 to 6, Table 5).

Configurations of major epoxides of α-methylstyrene were maintained in the epoxidations that were catalyzed by Mn1 to Mn5 (entries 7 to 12, Table 5). Furthermore, Mn1, Mn2, and Mn5 showed considerably higher e.e. values and moderate to high conversions for asymmetric epoxidation of α-methylstyrene (entries 7, 9, and 12, Table 5), obviously proposing the dominant role of (*R,R*)-salen in chiral configuration determination more than tartrate chirality. Next, catalytic activity of Mn2 (entry 9, Table 5), derived from the linkage of (*R,R*)-salen with (*S,S*)-tartrate, was comparable to the chiral sugar moiety-modified (salen)Mn(III) system [35], which may emphasize again that the additional chirality would affect chiral induction through coordination with Mn. On the other hand, catalytic epoxidation of indene gave excellent e.e. values, satisfactory conversions, as well as good TOFs, when salen and tartrate were configurationally identical (entries 19 and 23, Table 5). However, neither polymer catalysts (Mn1 to Mn4) nor monomeric Mn5 could increase chiral induction in epoxidation of *trans*-stilbene, and there are actually no e.e. values higher than 40% that are observed in all experiments (entries 13 to 18, Table 5).

Under homogeneous manner, Mn5 obviously exhibited more advantages than polymer catalysts in epoxidations of α-methylstyrene (entry 12, Table 5) and *trans*-stilbene (entry 18, Table 5). Mn1 showed comparatively higher e.e. values and acceptable to good conversions for conversions of styrene (entry 1, Table 5), α-methylstyrene (entry 7, Table 5), and indene (entry 19, Table 5). Mn4 gave a nice performance in transformation of indene too, which both emphasized the important role of uniform configuration that distributed between salen and tartrate.

### 3.11. Heterogeneous Catalysis from Zn/Al LDH-Supported (Tartrate-Salen)Mn(III)

Pure Zn/Al LDH and amino-LDH (3 mol% Al on alkene) had been respectively loaded as catalysts in asymmetric epoxidation of styrene, and neither Zn/Al LDH nor amino-LDH could give considerable epoxide products under TLC monitoring for 12 h, which meant (tartrate-salen)Mn(III) were catalytically active centers (entries 1 and 2, Table 6). Other catalytic reactions proceeded smoothly in the presence of either PhIO or *m*CPBA, but PhIO usually showed better e.e. values than *m*CPBA for almost all epoxidation reactions except for the case of indene catalyzed by Mn1@aL (entries 28 vs. 27, Table 6), probably due to a non-catalyzed way facilitated by *m*CPBA for epoxidation of alkenes [36].

Styrene was not an appropriate substrate for Mn1@aL to Mn4@aL, no excellent conversions and e.e. values were simultaneously detected for one single catalytic round (entries 3–10, Table 6), supposedly it was highly relative to a low enantiofacial selectivity in the transition state of catalytic styrene epoxidation [37]. Within this scope, Mn1@aL afforded 88% e.e. (*R*) and 29% conversion (entry 3, Table 6), while Mn3@aL afforded 69% e.e. (*S*) with 81% conversion (entry 7, Table 6), probably because the Zn-Al LDH matrix solidified a chiral-inducing environment for these two combinations. Similarly, there were no significant improvements in both conversion and enantioselectivity for epoxidation of *trans*-stilbene compared with homogeneous results (Table 4), and layered confinement was poor in promoting enantioselectivity (entries 19 to 26, Table 6). Even so, examples of identical chirality like Mn1@aL (entry 19, Table 6) and Mn4@aL (entry 25, Table 6) still showed better results than other endeavors.

Real progresses were found in epoxidation of α-methylstyrene, where Mn4@aL afforded a 97% conversion together with 93% e.e value under PhIO, but recycling failed (entry 17, Table 4). In the meantime, continuous high conversions and stable e.e. values appeared in the recycling catalyzed by Mn1@aL and Mn2@aL (entries 11 and 13, Table 6), being much better than their corresponding homogeneous catalysis (entries 7 and 9, Table 5), which was also equal or comparable to several heterogeneous systems [32,34]. Asymmetric epoxidation of indene succeeded too, when Mn3@aL had been loaded in association with PhIO, where conversions were maintained at a range of 69% to 91%, while e.e. values at 90% to 97%, showed potentials in large-scale production (entry 31, Table 6).

In this section, Mn1@aL looked like a powerful and steady heterogeneous catalyst for most alkenes in enantioselectivity as well as conversion. Mn3@aL should be second, but Mn2@aL and Mn4@aL were modest. Compared with homogeneous results (Table 5), identical chirality of salen and tartrate still offered positive chiral induction at this layered confinement.

## 4. Conclusions

A series of novel (tartrate-salen)Mn(III) polymer catalysts were prepared and employed in asymmetric epoxidations of unfunctionalized alkenes under both homogeneous and heterogeneous manners, which revealed chiral synergetic effects of salen chirality with tartrate linker chirality. In addition to comprehensive characterizations of the synthesized intermediates and products, scanning electron microscopy revealed an interesting morphological change during chemical modification of Zn/Al LDH surfaces. Other characterizations including nitrogen physisorption and powdered XRD indicated Mn polymers were dispersed into LDH. On the other hand, when salen unit and tartrate linker showed identical configurations, the enhanced enantioselectivity could be obtained. Herein, Mn1, a combination of (*R,R*)-tartrate with (*R,R*)-(salen)Mn(III), along with its heterogeneous catalysts, showed the best catalytic performance involving chiral induction, activity, as well as stability. In addition, α-methylstyrene and indene appeared to be appropriate substrates in this system, but *trans*-stilbene and styrene showed little credits as substrates.

## Figures and Tables

**Table 1:** Textural and physicochemical properties of the synthesized samples.

Sample	*S*_BET_^a^	PV ^b^	PR ^c^	*ρ* ^d^	*d*_S_^e^	*d*_W_^f^	*ζ* ^g^
Zn/Al LDH	11.03	7.9 × 10^−2^	119.4	1.20	453.3	109.7	−25.51
Mn1@aL	4.93	3.6 × 10^−2^	140.5	0.90	1352.2	362.7	2.39

^a^ Surface area (m^2^ g^−1^) determined by BET method based on N_2_ adsorption. ^b^ Pore volume (cm^3^ g^−1^), BJH method on N_2_ adsorption. ^c^ Pore radius (nm), BJH method on N_2_ adsorption. ^d^ Bulk density (g cm^−3^). ^e^ Crystallite size (nm) based on BET surface area: *d*_S_ = 6/(*S*_BET_·*ρ*), *ρ* bulk density. ^f^ Diameter of particle in CH_2_Cl_2_ (nm). ^g^ Zeta potential of particle in CH_2_Cl_2_ (mV).

**Table 2:** Chemical composition data of synthetic samples.

Sample	Elemental Analysis ^a^			Zn ^b^	Al ^b^	Mn ^b^	N/Mn ^c^
	**C**	**H**	**N**				
L1	66.8 (67.1)	6.7 (7.2)	5.5 (4.6)	-	-	-	-
Mn1	56.3 (55.7)	6.1 (6.2)	4.5 (3.8)	-	-	1.10 (1.36)	2.9
LDH	1.6 (1.3)	2.9 (3.0)	-	6.77 (6.21)	2.21 (2.05)	-	-
amino-LDH	17.5 (17.2)	3.1 (2.8)	0.8 (0.8)	6.95 (6.03)	1.70 (1.99)	-	-
Mn1@aL	20.3 (21.6)	2.6 (3.2)	1.1 (1.1)	6.60 (5.46)	2.22 (1.77)	0.18 (0.15)	4.3

^a^ Weight percentage (wt%), data shown in parentheses were calculated values. ^b^ Determined by ICP-AES (mmol g^−1^), data shown in parentheses were calculated values. ^c^ Molar ratio of N to Mn, deduced from N% found and Mn content detected.

**Table 3:** Binding energies and surface atomic compositions of C, N, Mn, Zn, and Al species in synthetic samples.

Sample	C (1s)	Si (2p)	N (1s)	Mn (2p)	Zn (2p)	Al (2p)
Zn/Al LDH	282.0 (57.1) ^a^	-	397.0 (3.3)	-	1019.0 (1.5)	71.0 (3.0)
amino-LDH	282.0 (49.1)	99.0 (3.5)	397.0 (3.4)	-	1019.0 (2.1)	71.0 (4.3)
Mn1@aL	282.0 (84.9)	99.0 (0.6)	396.0 (0.5)	638.0 (0.4)	1019.0 (0.07)	-

^a^ Binding energy (eV), together with atomic percentage (at%) in parentheses.

**Table 4:** Asymmetric epoxidation of styrene for study of catalytic conditions.

Entry ^a^	Catalyst ^b^	Solvent ^c^	Conversion ^d^ (%)	E.e. ^e^ (%)	TOF ^f^
1	3	Acetone	22	16 (*R*)	1.2
2	3	Diethyl ether	36	13 (*R*)	2.0
3	3	Acetonitrile	33	3 (*R*)	1.8
4	3	*n*-Hexane	40	19 (*R*)	2.2
5	3	Ethanol	21	11 (*R*)	1.1
6	3	Water	8	7 (*R*)	0.8
7	3	Dichloromethane	11	38 (*R*)	0.6
8	1.5	Dichloromethane	10	19 (*R*)	0.5
9	6	Dichloromethane	26	21 (*R*)	1.4

^a^ Conditions (as in Section 2.2): styrene (1 mmol), ammonium acatate (co-catalyst, 0.12 mmol), iodobenzene (PhIO, 1 mmol), solvent (5 mL), *T* (273 K), time (6 h). ^b^ Mn1 loading amount, based on the molar ratio of manganese content to alkene substrate (mol%). ^c^ 5 mL. ^d^ Molar ratio of epoxide products to alkene, determined by chiral HPLC. ^e^ Enantiomeric excess, determined by chiral HPLC. Major epoxides of all entries were (*R*)-styrene oxide, based on comparison with literature data [33] (Section S5, Supplementary data). ^f^ Turnover frequency, h^−1^.

**Table 5:** Asymmetric epoxidation of unfunctionalized alkenes catalyzed by homogeneous (tartrate-salen)Mn(III) polymer catalysts.

Entry ^a^	Alkene	Product	Catalyst ^b^	Oxidant ^c^	Conv. ^d^ (%)	E.e. ^e^ (%)	TOF ^f^
1 ^a^	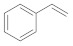	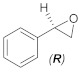 and 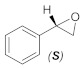	Mn1	PhIO	11	38 (R)	0.6
2			Mn1	*m*CPBA	77	12 (R)	4.2
3			Mn2	PhIO	25	16 (R)	1.3
4			Mn3	PhIO	19	36 (S)	1.0
5			Mn4	PhIO	26	21 (S)	1.4
6			Mn5	PhIO	67	30 (R)	3.7
7	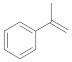	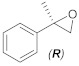 and 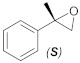	Mn1	PhIO	78	49 (R)	4.3
8			Mn1	*m*CPBA	80	10 (R)	4.4
9			Mn2	PhIO	81	61 (R)	4.5
10			Mn3	PhIO	17	9 (R)	0.9
11			Mn4	PhIO	32	8 (R)	1.7
12			Mn5	PhIO	22	77 (R)	1.2
13	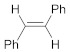	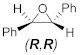 and 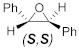	Mn1	PhIO	54	5 (R,R)	3.0
14			Mn1	*m*CPBA	55	10 (R,R)	3.0
15			Mn2	PhIO	31	20 (R,R)	1.7
16			Mn3	PhIO	41	5 (S,S)	2.2
17			Mn4	PhIO	25	11 (S,S)	1.3
18			Mn5	PhIO	92	33 (R,R)	5.1
19	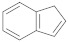	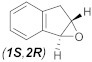 and 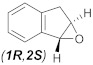	Mn1	PhIO	36	67 (1S,2R)	2.0
20			Mn1	*m*CPBA	61	55 (1S,2R)	3.3
21			Mn2	PhIO	9	26 (1S,2R)	0.5
22			Mn3	PhIO	100	44 (1R,2S)	5.5
23			Mn4	PhIO	100	78 (1R,2S)	5.5
24			Mn5	PhIO	98	39 (1S,2R)	5.4

^a^ Conditions (as in Section 2.2): alkene (1 mmol), ammonium acetate (co-catalyst, 0.12 mmol), CH_2_Cl_2_ (5 mL), *T* (273 K), time (6 h). Entry 1 in this table was identical to entry 7 of Table 4. ^b^ Catalyst loading, based on molar ratio of Mn to alkene (3 mol%). ^c^ PhIO or *m*CPBA, 1 mmol. ^d^ As in Table 4. ^e^ As in Table 4, for styrene oxide [33]. In addition, major epoxide configurations of α-methylstyrene oxide [33], *trans*-stilbene oxide [33], and indene oxide [34] were determined by chiral HPLC, after comparison with literatures (Section S5, Supplementary data). ^f^ As in Table 4.

**Table 6:** Asymmetric epoxidation of unfunctionalized alkenes catalyzed by Zn/Al LDH-supported (tartrate-salen)Mn(III) polymers.

Entry ^a^	Alkene	Catalyst ^b^	Oxidant ^c^	Conversion ^d^ (%)	E.e. ^f^ (%)	TOF ^g^
1	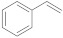	Zn/Al LDH	PhIO	N.f.^e^	-	-
2		amino-LDH	PhIO	N.f.	-	-
3		Mn1@aL	PhIO	29	88 (R)	1.6
4		Mn1@aL	*m*CPBA	70	16 (R)	3.8
5		Mn2@aL	PhIO	32	56 (R)	1.7
6		Mn2@aL	*m*CPBA	79	6 (R)	4.3
7		Mn3@aL	PhIO	81	69 (S)	4.5
8		Mn3@aL	*m*CPBA	57	45 (S)	3.1
9		Mn4@aL	PhIO	55	10 (R)	3.0
10		Mn4@aL	*m*CPBA	90	5 (R)	5.0
11	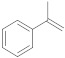	Mn1@aL	PhIO	75 (86, 80, 65,62)	71 (75, 77, 52, 49)/(R)	4.1
12		Mn1@aL	*m*CPBA	81	12 (R)	4.5
13		Mn2@aL	PhIO	83 (89, 90, 86, 75)	64 (61, 61, 60, 51)/(R)	4.6
14		Mn2@aL	*m*CPBA	67	13 (R)	3.7
15		Mn3@aL	PhIO	55	29 (R)	3.0
16		Mn3@aL	*m*CPBA	89	25 (S)	4.9
17		Mn4@aL	PhIO	97 (96)	93 (0.2)/(S)	5.3
18		Mn4@aL	*m*CPBA	90	57 (S)	5.0
19	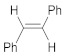	Mn1@aL	PhIO	28	12 (R,R)	1.5
20		Mn1@aL	*m*CPBA	38	0.6 (S,S)	2.1
21		Mn2@aL	PhIO	32	9 (R,R)	1.7
22		Mn2@aL	*m*CPBA	33	1 (S,S)	1.8
23		Mn3@aL	PhIO	26	10 (S,S)	1.4
24		Mn3@aL	*m*CPBA	19	5 (S,S)	1.0
25		Mn4@aL	PhIO	44	18 (S,S)	2.4
26		Mn4@aL	*m*CPBA	40	1 (S,S)	2.2
27	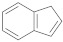	Mn1@aL	PhIO	82	69 (1S,2R)	4.5
28		Mn1@aL	*m*CPBA	65	71 (1S,2R)	3.6
29		Mn2@aL	PhIO	51	81 (1S,2R)	2.8
30		Mn2@aL	*m*CPBA	61	60 (1S,2R)	3.3
31		Mn3@aL	PhIO	85 (85, 80, 91, 69)	91 (98, 93, 97, 90)/(1R,2S)	4.7
32		Mn3@aL	*m*CPBA	96	29 (1R,2S)	5.3
33		Mn4@aL	PhIO	74	71 (1R,2S)	4.1
34		Mn4@aL	*m*CPBA	89	70 (1R,2S)	4.9

^a^ Conditions (as in Section 2.2): alkene (1 mmol), ammonium acetate (co-catalyst, 0.12 mmol), CH_2_Cl_2_ (5 mL), *T* (273 K), time (6 h). ^b^ Catalyst loading, based on the molar ratio of Mn content to alkene (3 mol%). ^c^ PhIO or *m*CPBA, 1 mmol. ^d^ As in Table 3, and data in parentheses represented consecutively recycled results. ^e^ No epoxides were found on HPLC. ^f^ As in Table 5, and data in parentheses represented consecutively recycled results along with configurations of major epoxides throughout recycling (Section S5, Supplementary data). ^g^ Turnover frequency of cycle fresh, h^−1^.

## Supplementary Materials

The following are available online at https://www.mdpi.com/1996-1944/13/21/4860/s1, Section S1: Characterization of bis-aldehyde (3b); Section S2. Characterization of chiral polymeric salen ligands (L2, L3, and L4); Section S3. Characterization of (tartrate-salen)Mn(III) polymers (Mn2, Mn3, and Mn4); Section S4. Characterization of amino-functionalized Zn(II)/Al(III) LDH-supported (tartrate-salen)Mn(III) polymers (Mnx@aL, x = 2, 3, 4); Section S5. HPLC separation details and representative chromatograms
