# Supplementary material for: Oligomeric (Salen)Mn(III) Complexes Featuring Tartrate Linkers Immobilized over Layered Double Hydroxide for Catalytically Asymmetric Epoxidation of Unfunctionalized Olefins
Source: Materials (Basel). 2020 Oct 29;13(21):4860. doi: 10.3390/ma13214860 (PMC7672564; doi:10.3390/ma13214860)
Supplement: Supplementary file 1 [file materials-13-04860-s001.zip › materials-889194-supplementary.docx]

**Supplementary Materials**

Oligomeric (Salen)Mn(III) Complexes Featuring Tartrate Linkers Immobilized over Layered Double Hydroxide for Catalytically Asymmetric Epoxidation of Unfunctionalized Olefins

Yihong Jia 1, Asma A. ALOthman 2, Rui Liang 3, Xiaoyong Li 1, Weiyi Ouyang 1, Xiangdong Wang 1, Yong Wu 1, Sameh M. Osman 2, Zhaorui Li 4, Min Gao 1,* and Yang Sun 1,*

1 Department of Applied Chemistry, School of Science, Xi’an Jiaotong University, No. 28, Xianning West Road, Xi’an 710049, China; [jiayihong@stu.xjtu.edu.cn](mailto:jiayihong@stu.xjtu.edu.cn) (Y.J.); lixy6658@mail.xjtu.edu.cn (X.L.); weyi.ouyang@gmail.com (W.O.); wang90xd@163.com (X.W.); specwy@mail.xjtu.edu.cn (Y.W.)

2 Department of Chemistry, College of Science, King Saud University, P.O. Box 2455, Riyadh 11451, Saudi Arabia; [aaalothman@ksu.edu.sa](mailto:aaalothman@ksu.edu.sa) (A.A.A.); smahmoud@ksu.edu.sa (S.M.O.)

3 School of Resources and Environment, Anhui Agriculture University, No. 130, Changjiang West Road, Hefei 230036, China; liangrui@ahau.edu.cn

4 College of Materials Science and Engineering, Xi’an University of Science and Technology, No. 48, Shaangu Avenue, Lintong District, Xi’an 710600, China; lzr981013@163.com

***** Correspondence: gaominxjtu@mail.xjtu.edu.cn (M.G.); sunyang79@mail.xjtu.edu.cn (Y.S.). Tel.: +86-29-82663914; Fax: +86-29-82668559(Y.S.)

**Table of Contents**

**S1**. Characterization of bis-aldehyde (3b)

**S2.** Characterization of chiral polymeric salen ligands (L2, L3, and L4)

**S3.** Characterization of (tartrate-salen)Mn(III) polymers (Mn2, Mn3, and Mn4)

**S4.** Characterization of amino-functionalized Zn(II)/Al(III) LDH-supported (tartrate-salen)Mn(III) polymers (Mn*x*@aL, *x* = 2, 3, 4)

**S5**. HPLC separation details and representative chromatograms

Section

S1. Characterization of bis-aldehyde (3b)

3b, yellow solid, 1.70 g, 44% yield. 1H NMR (400 MHz, CDCl3) δH, ppm: 1.28 (9H, s, C(C*H*3)3), 3.93 (4H, s, C*H*2 on methylene), 4.59 (2H, s, C*H* on tartrate), 7.20-7.25 (2H, m, Ar*H*), 7.50-7.59 (2H, m, Ar*H*), 9.85 (2H, s, C*H*O). FT-IR (KBr) σ, cm-1: 3430 (m, O-H on tartrate), 3395-3230 (br, s, ArO-H), 2936 (s, C-H on methyl), 2856 (s, C-H on methylene), 1722 (vs, C=O on salicylaldehyde). ESI-HRMS (positive, m/z): 553.5763 (Calcd. for [M+Na]+ 553.5519). = +11 (*c* 0.01 g mL-1, CH2Cl2). Ideal formula of **3b** is C28H34O10. Anal. Calcd.: C, 63.4; H, 6.4. Found: C, 63.1; H, 6.7.

S2. Characterization of chiral polymeric salen ligands (L2, L3, and L4)

L2, 1H NMR (400 MHz, CDCl3) δH, ppm: 1.40 (9H, s, C(C*H*3)3), 1.42 – 1.57 (8H, m, C*H*2 on cyclohexyl), 2.33 (2H, s, C*H* on cyclohexyl), 3.91 (4H, s, C*H*2 on methylene), 4.50 (2H, s, C*H* on tartrate), 7.35-7.40 (2H, m, Ar*H*), 7.50-7.54 (2H, m, Ar*H*), 9.89 (2H, s, C*H*N). FT-IR (KBr) σ, cm-1: 3442 (br, s, O-H on tartrate, ArO-H, overlapped), 2963 and 2865 (both m, C-H on methyl), 2929 (both m, C-H on methylene), 1772 (w, tartrate), 1648 (s, C=N), 1558 (w, C-O), 1268 (w, Ar-OH). = +309 (*c* 0.01 g mL-1, CH2Cl2). *M*n = 8512, *M*w = 10214, PDI (*M*w/*M*n) = 1.2. Based on *M*n, number of tartrate-salen monomers was 14.0, ideal formula of L2 was deduced as (C4H4O6·C30H40O2N2)14.0. Anal. Calcd.: C, 67.1; H, 7.2; N, 4.6. Found: C, 67.2; H, 6.5; N, 5.5.

L3, 1H NMR (400 MHz, CDCl3) δH, ppm: 1.41 (9H, s, C(C*H*3)3), 1.40 - 1.60 (8H, m, C*H*2 on cyclohexyl), 2.34 (2H, s, C*H* on cyclohexyl), 3.90(4H, s, C*H*2 on methylene), 4.51 (2H, s, C*H* on tartrate), 7.36-7.39 (2H, m, Ar*H*), 7.51-7.54 (2H, m, Ar*H*), 9.88 (2H, s, C*H*N). FT-IR (KBr) σ, cm-1: 3443 (br, s, O-H on tartrate, ArO-H, overlapped), 2964 and 2868 (m, C-H on methyl), 2930 (m, C-H on methylene), 1772 (w, tartrate), 1649 (s, C=N), 1558 (w, C-O), 1268 (w, Ar-OH). = -229 (*c* 0.01 g mL-1, CH2Cl2). *M*n = 7843, *M*w = 11764, PDI (*M*w/*M*n) = 1.5. Based on *M*n, number of tartrate-salen monomers was 12.9, ideal formula of L3 was deduced as (C4H4O6·C30H40O2N2)12.9. Anal. Calcd.: C, 67.1; H, 7.2; N, 4.6. Found: C, 68.9; H, 7.2; N, 5.7.

L4, 1H NMR (400 MHz, CDCl3) δH, ppm: 1.40 (9H, s, C(C*H*3)3), 1.42 – 1.57 (8H, m, C*H*2 on cyclohexyl), 2.33 (2H, s, C*H* on cyclohexyl), 3.91 (4H, s, C*H*2 on methylene), 4.50 (2H, s, C*H* on tartrate), 7.35-7.40 (2H, m, Ar*H*), 7.50-7.54 (2H, m, Ar*H*), 9.89 (2H, s, C*H*N). FT-IR (KBr) σ, cm-1: 3444 (br, s, O-H on tartrate, ArO-H, overlapped), 2962 and 2868 (both m, C-H on methyl), 2933 (m, C-H on methylene), 1772 (w, tartrate), 1649 (s, C=N), 1558 (w, C-O), 1268 (w, Ar-OH). = -70 (*c* 0.01 g mL-1, CH2Cl2). *M*n = 6019, *M*w = 12639, PDI (*M*w/*M*n) = 2.1. Based on *M*n, the number of tartrate-salen monomers was 9.9, ideal formula of L4 was deduced as (C4H4O6·C30H40O2N2)9.9. Anal. Calcd.: C, 67.1; H, 7.2; N, 4.6. Found: C, 68.6; H, 8.1; N, 4.5.

S3. Characterization of (tartrate-salen)Mn(III) polymers (Mn2, Mn3, and Mn4)

Mn2, brown powders (0.81 g), FT-IR (KBr) σ, cm–1: 3440 (br, s, O-H on tartrate, H-OH, overlapped), 2959 and 2866 (both m, C−H on methyl), 2931 (m, C–H on methylene), 1740 (w, tartrate), 1632 (s, C=N), 1268 (w, Ar–OMn). = +332 (*c* 0.01 g mL−1, CH2Cl2). Based on L2, ideal formula of Mn2 was summarized as (C4H4O6·C30H38O2N2MnCl·H2O)14.0. Anal. Calcd.: C, 57.1; H, 6.1; N, 3.9. Found: C, 55.3; H, 6.2; N, 4.9. Mn3+ is 1.20 mmol g−1 determined by ICP-AES.

Mn3, brown powders (0.69 g), FT-IR (KBr) σ, cm-1: 3443 (br, s, O–H on tartrate), 2958 and 2868 (both m, C–H on methyl), 2932 (m, C-H on methylene), 1735 (w, tartrate), 1635 (s, C=N), 1558 (w, C–O), 1268 (w, Ar–OMn), 571 (w, Mn-O). = −66 (*c* 0.01 g mL−1, CH2Cl2). Based on L3, ideal formula of Mn3 was summarized as (C4H4O6·C30H38O2N2MnCl)12.9. Anal. Calcd.: C, 58.5; H, 6.0; N, 4.0. Found: C, 56.6; H, 6.9; N, 3.9. Mn3+ is 1.39 mmol g−1 determined by ICP-AES.

Mn4, brown powders (0.85 g), FT-IR (KBr) σ, cm−1: 3437 (br, s, O-H on tartrate, H–OH, overlapped), 2957 and 2868 (both m, C–H on methyl), 2933 (m, C-H on methylene), 1645 (s, C=N), 1267 (w, Ar–OMn), 568 (w, Mn–O). = −185 (*c* 0.01 g mL−1, CH2Cl2). Based on L4, ideal formula of Mn4 was summarized as (C4H4O6·C30H38O2N2MnCl·3H2O)9.9. Anal. Calcd.: C, 54.3; H, 6.3; N, 3.7. Found: C, 55.7; H, 6.3; N, 4.6. Mn3+ is 1.06 mmol g-1 determined by ICP-AES.

S4. Characterization of amino-functionalized Zn(II)/Al(III) LDH-supported (tartrate-salen)Mn(III) polymers (Mnx@aL, x = 2, 3, 4)

Mn2@aL, yellowish-brown powders (0.51 g). FT-IR (KBr) σ, cm−1: 3708 and 3673 (both w, AlO–H), 3649 and 3588 (both m, ZnO–H), 3443 (br, s, O−H on tartrate, N–H, overlapped), 2961 and 2869 (both w, C−H on methyl), 2925 (w, C-H on methylene), 1770 (w, tartrate), 1699 (m, C=O on benzoate), 1631 (m, C=N), 1558 (m, C–O on tartrate), 1261 (w, Ar–O), 1130 (s, Si–C), 877 (w, Si–O), 567 (w, Mn–O), and 419 (m, Mn–N). Ideal formula of Mn2@aL was [Zn2.09Al0.69(OH)5.23]1.00[C6H5COO]0.62[C3H8NO3Si]0.21[C4H4O6·C30H38O2N2MnCl]0.12. Anal. Calcd.: C, 25.2; H, 3.4; N, 1.4. Found: C, 27.0; H, 4.7; N, 1.4. Mn3+, Zn2+, and Al3+ were 0.27, 4.85, and 1.60 mmol g-1 based on ideal formula, while 0.30, 4.94, and 1.77 mmol g−1 under ICP-AES detection.

Mn3@aL, yellowish-brown powders (0.29 g). FT-IR (KBr) σ, cm−1: 3708 and 3673 (both w, AlO–H), 3649 and 3587 (both m, ZnO–H), 3442 (br, s, O-H on tartrate, N-H, overlapped), 2960 and 2871 (both w, C–H on methyl), 2925 (w, C−H on methylene), 1770 (w, tartrate), 1699 (m, C=O on benzoate), 1631 (m, C=N), 1559 (m, C–O on tartrate), 1261 (w, Ar–O), 1132 (s, Si–C), 568 (m, Mn–O), and 419 (w, Mn–N). Ideal formula of Mn3@aL was [Zn2.09Al0.69(OH)5.23]1.00[C6H5COO]0.62[C3H8NO3Si]0.21[C4H4O6·C30H38O2N2MnCl]0.08. Anal. Calcd.: C, 22.9; H, 3.3; N, 1.2. Found: C, 21.6; H, 4.0; N, 1.2. Mn3+, Zn2+, and Al3+ were 0.19, 5.19, and 1.71 mmol g−1 based on ideal formula, while 0.22, 4.65, and 1.98 mmol g−1 under ICP-AES detection.

Mn4@aL, brown powders (0.50 g). FT-IR (KBr) σ, cm−1: 3748 and 3673 (both w, AlO–H), 3648 (w, ZnO−H), 3447 (br, s, O–H on tartrate, N–H, overlapped), 2945 and 2870 (both w, C–H on methyl), 2927 (w, C–H on methylene), 1734 (w, tartrate), 1699 (m, C=O on benzoate), 1605 (m, C=N), 1557 (w, C–O on tartrate), 1139 (s, Si–C), and 420 (m, Mn–N). Ideal formula of Mn4@aL was [Zn2.09Al0.69(OH)5.23]1.00[C6H5COO]0.62[C3H8NO3Si]0.21[C4H4O6·C30H38O2N2MnCl]0.07. Anal. Calcd.: C, 22.3; H, 3.2; N, 1.2. Found: C, 23.6; H, 3.2; N, 0.6. Mn3+, Zn2+, and Al3+ were 0.17, 5.28, and 1.74 mmol g−1 based on ideal formula, while 0.16, 5.92, and 2.05 mmol g−1 under ICP-AES detection.

S5. HPLC separation details and representative chromatograms

5.1. HPLC separation conditions

As shown in 2.2 Characterization of 2. Experimental.

5.2. Determination of epoxide enantiomers

To recognize chromatographic behaviors of epoxide enantiomers:

1. chromatographically pure styrene, α-methylstyrene, *trans*-stilbene, and indene were tested on the present HPLC, to yield their corresponding retention times;
2. racemic epoxides of four alkenes were prepared by a non-chiral [*N,N’*-bis(3,5-di-*tert*-butylsalicylidene)-ethylenediaminato(2-)]manganese(III)chloride under buffered NaClO [a], and further tested on same HPLC to obtain retention times;
3. non-racemic epoxide samples of styrene [b], α-methylstyrene [b], *trans*-stilbene [b], and indene [c] were prepared according to literatures, and tested on same HPLC;

Comparing results from **1)**, **2)**, **3)**, together with the reported configuration of major epoxide products [b,c], each configuration along with retention time was confirmed.

Ref.:

1. E.N. Jacobsen, W. Zhang, A.R. Muci, J.R. Ecker, L. Deng. Highly enantioselective epoxidation catalysts derived from 1,2-diaminocyclohexane. *J. Am. Chem. Soc.* **1991**, *113,* 7063–7064.
2. W. Zhang, J.L. Loebach, S.R. Wilson, E.N. Jacobsen. Enantioselective epoxidation of unfunctionalized olefins catalyzed by (salen)manganese complexes. *J. Am. Chem. Soc.* **1990**, *112*, 2801–2803.
3. C.E. Song, E.J. Roh. Practical method to recycle a chiral (salen)Mn epoxidation catalyst by using an ionic liquid. *Chem. Commun.* **2000**, 837–838.

5.3. Representative chromatograms

Part 1. Asymmetric epoxidation of styrene

(1) pure styrene


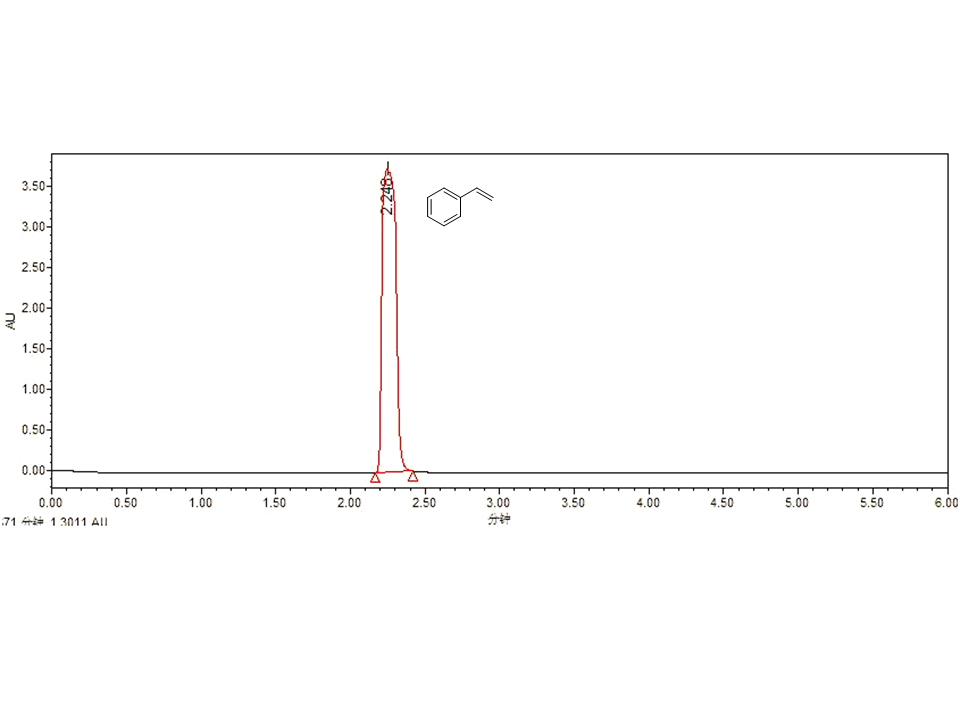


(2) racemic styrene oxide


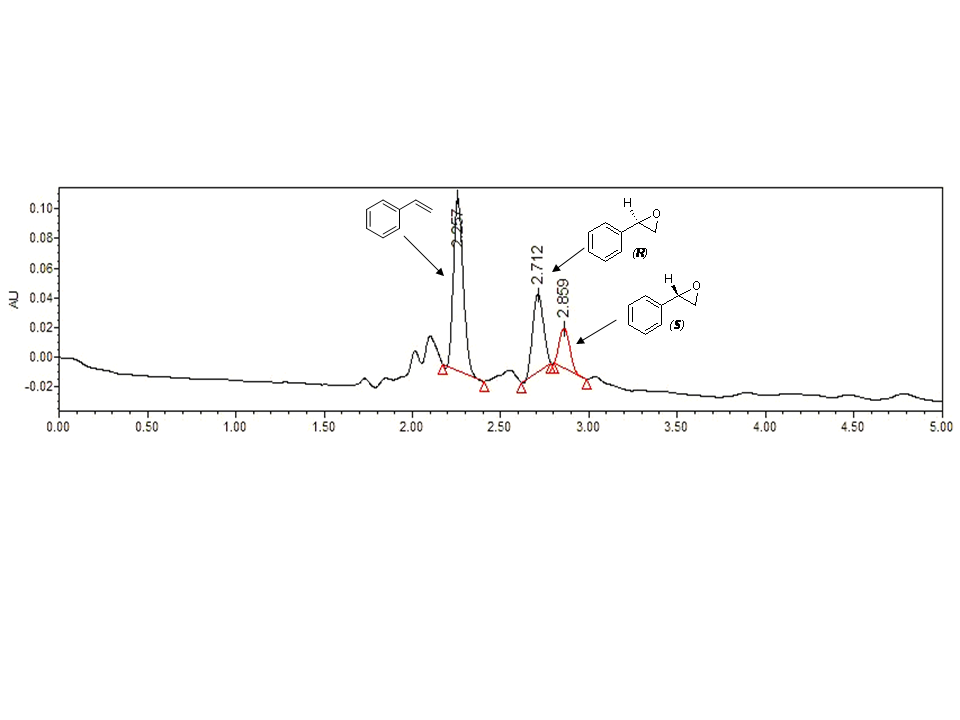


Chromatographic data of racemic styrene oxide:

| **Retention Time**  **(min)** | **Area**  **(μV·s)** | **% Area** | **Height**  **(μV)** | **Integration Type** |
| --- | --- | --- | --- | --- |
| 2.257 | 448,175 | 57.49 | 116,435 | bb |
| 2.712 | 222,663 | 28.56 | 52,345 | bb |
| 2.859 | 108,740 | 13.95 | 26,645 | bb |

(3) Entry 7, Table 4


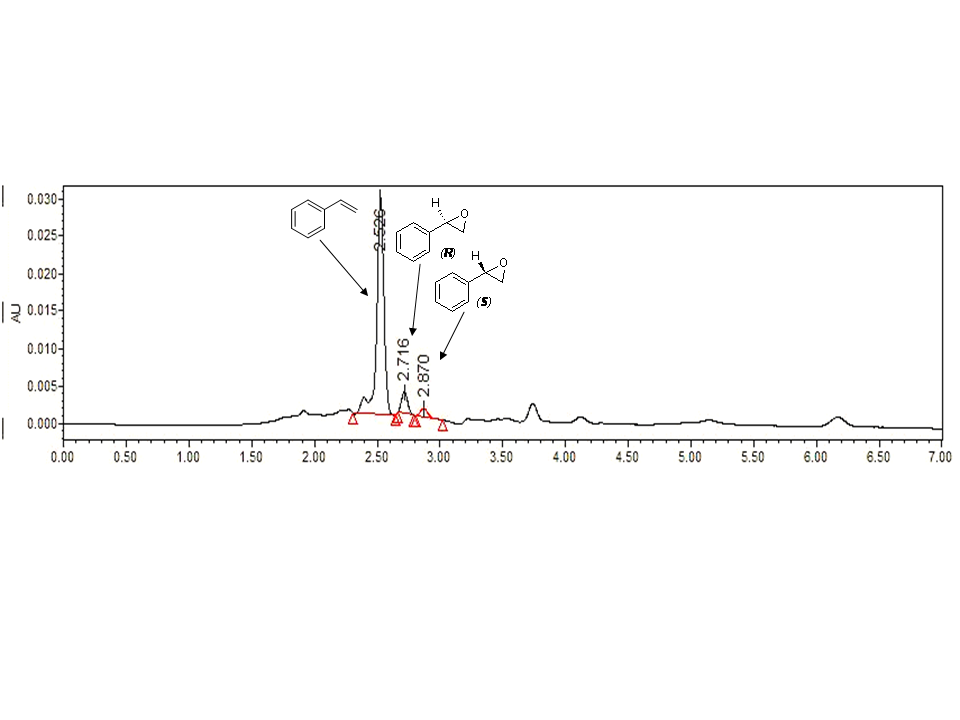


| **Retention Time**  **(min)** | **Area**  **(μV·s)** | **% Area** | **Height**  **(μV)** | **Integration Type** |
| --- | --- | --- | --- | --- |
| 2.526 | 117,385 | 89.21 | 28,949 | bb |
| 2.716 | 9843 | 7.48 | 2863 | bb |
| 2.870 | 4355 | 3.31 | 1118 | bb |

E.e. (%) = 38% (R); Conv. (%) = 11%.

Part 2. Asymmetric epoxidation of α-methylstyrene

(1) pure α-methylstyrene


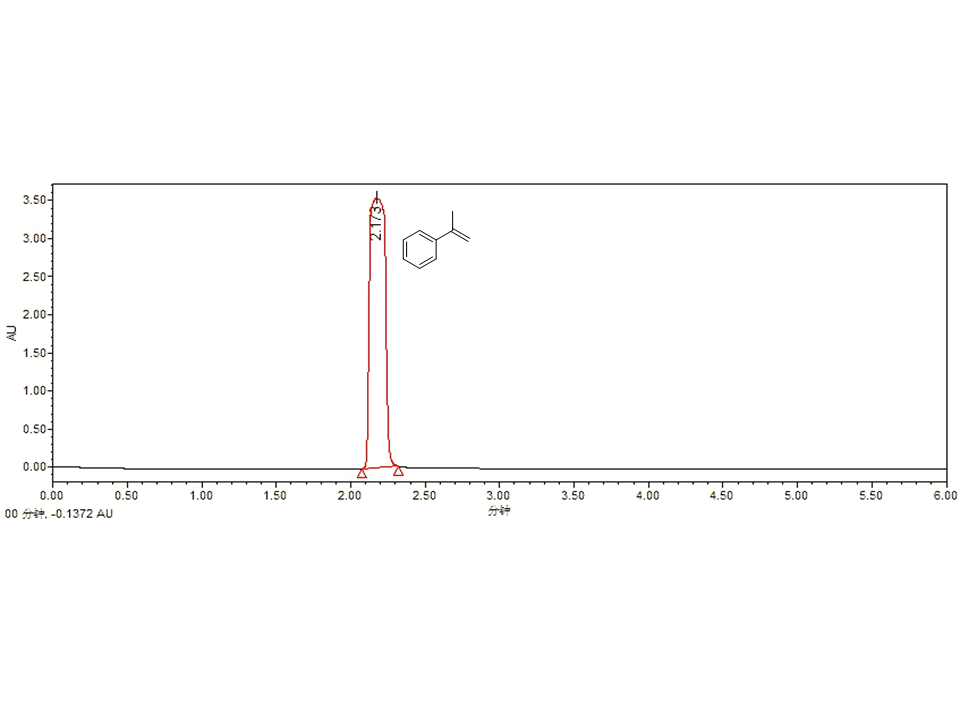


(2) racemic α-methylstyrene oxide


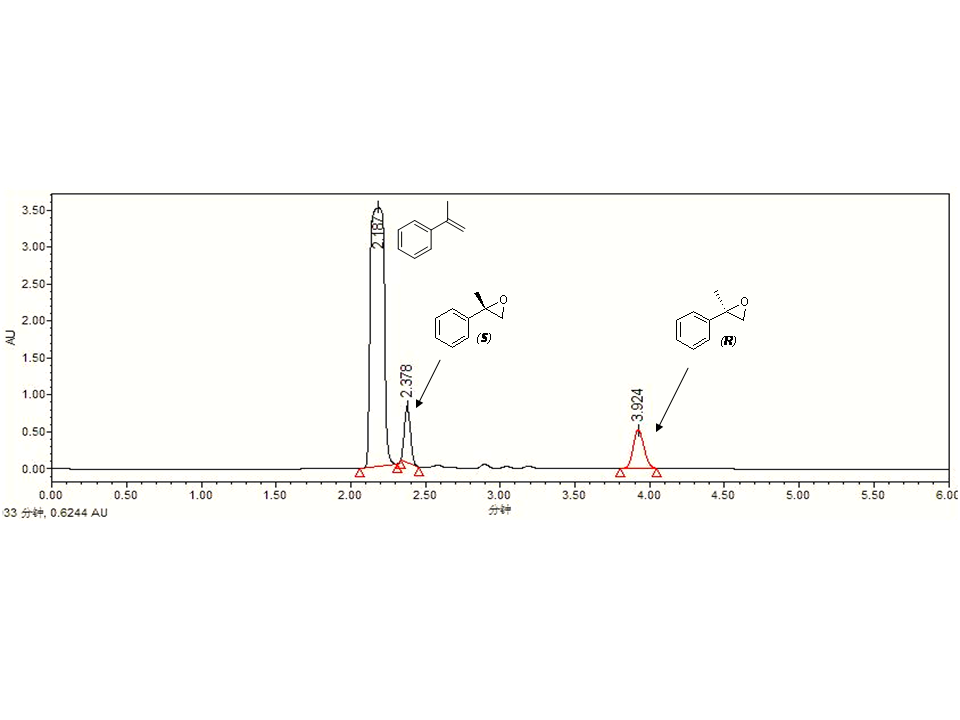


Chromatographic data of racemic α-methylstyrene oxide:

| **Retention Time**  **(min)** | **Area**  **(μV·s)** | **% Area** | **Height**  **(μV)** | **Integration Type** |
| --- | --- | --- | --- | --- |
| 2.187 | 21,844,858 | 82.30 | 3,502,715 | bb |
| 2.378 | 2,120,623 | 7.99 | 761,762 | bb |
| 3.924 | 2,577,458 | 9.71 | 516,939 | bb |

(3) Entry 7, Table 5


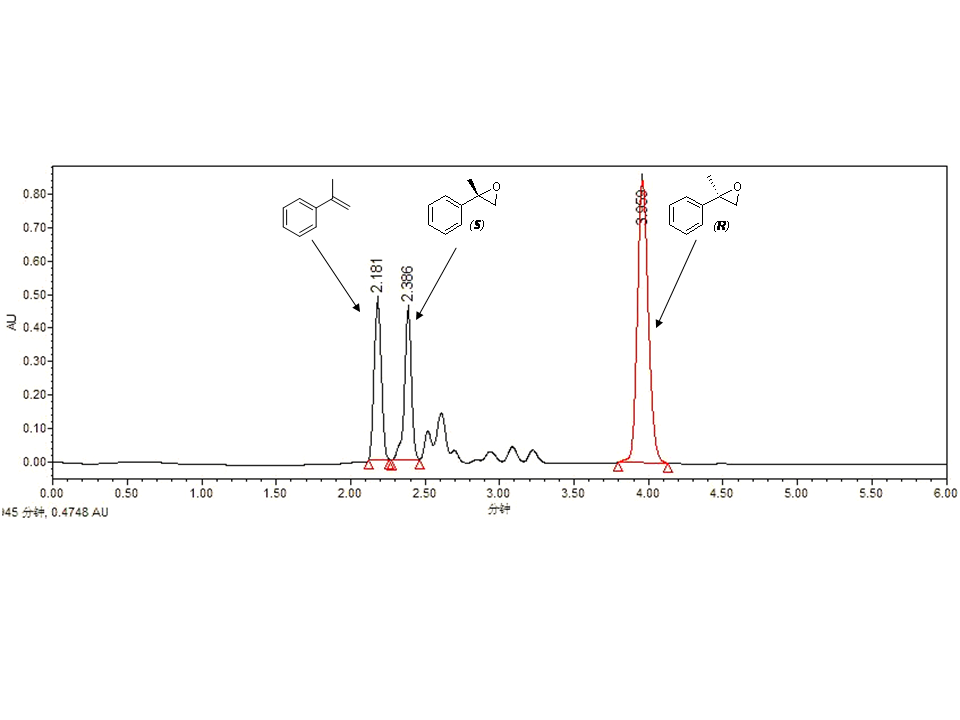


Chromatographic data:

| **Retention Time**  **(min)** | **Area**  **(μV·s)** | **% Area** | **Height**  **(μV)** | **Integration Type** |
| --- | --- | --- | --- | --- |
| 2.181 | 1,582,663 | 21.57 | 470,290 | bb |
| 2.386 | 1,459,617 | 19.89 | 445,416 | bb |
| 3.959 | 4,296,668 | 58.55 | 842,467 | bb |

E.e. (%) = 49% (R); Conv. (%) = 78%.

(4) Entry 11, Table 5


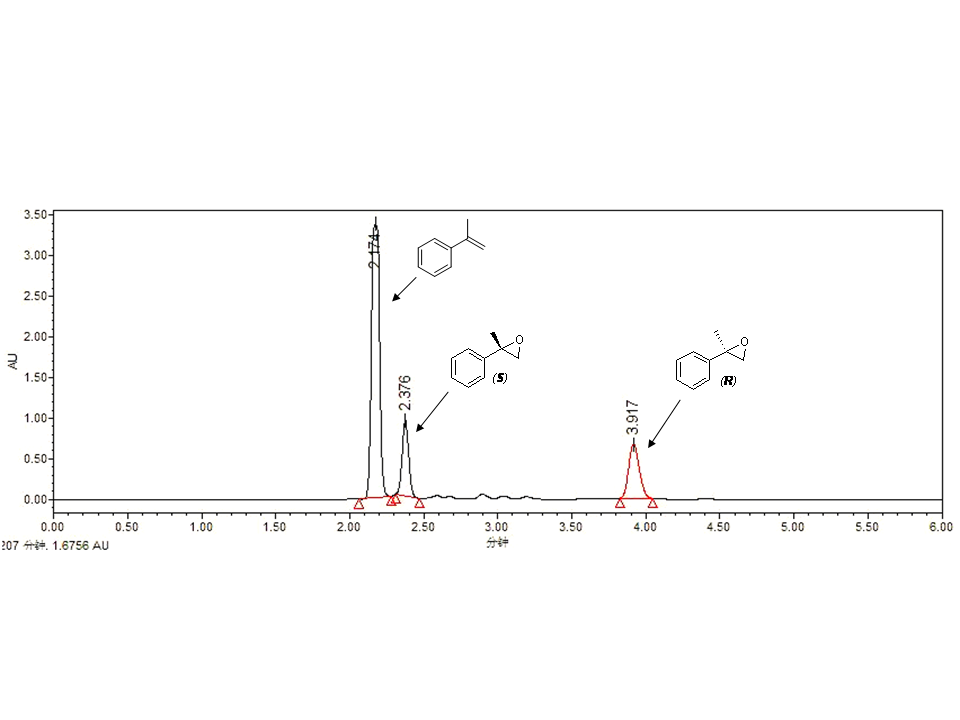


Chromatographic data:

| **Retention Time**  **(min)** | **Area**  **(μV·s)** | **% Area** | **Height**  **(μV)** | **Integration Type** |
| --- | --- | --- | --- | --- |
| 2.174 | 12,726,559 | 67.53 | 3,366,923 | bb |
| 2.376 | 2,809,414 | 14.91 | 933,328 | bb |
| 3.917 | 3,309,325 | 17.56 | 666,972 | bb |

E.e. (%) = 8% (R); Conv. (%) = 32%.

(5) Entry 12, Table 5


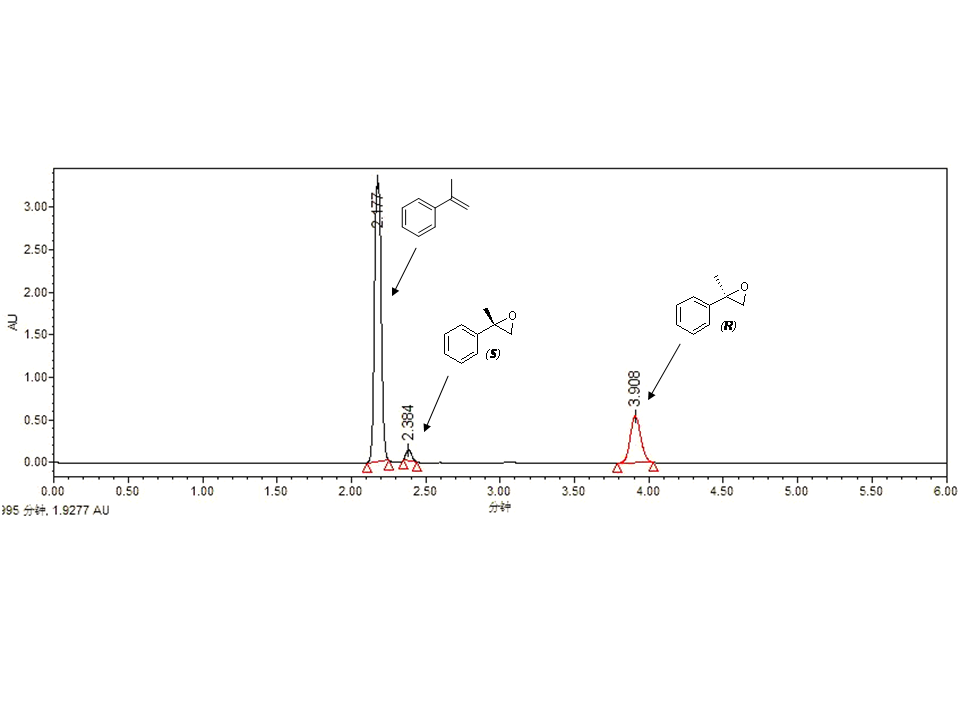


Chromatographic data:

| **Retention Time**  **(min)** | **Area**  **(μV·s)** | **% Area** | **Height**  **(μV)** | **Integration Type** |
| --- | --- | --- | --- | --- |
| 2.177 | 10,170,673 | 77.15 | 3,277,032 | bb |
| 2.384 | 333,954 | 2.53 | 126,592 | bb |
| 3.908 | 2,678,613 | 20.32 | 547,814 | bb |

E.e. (%) = 77% (R); Conv. (%) = 22%.

(6) Last circle of Entry 11, Table 6


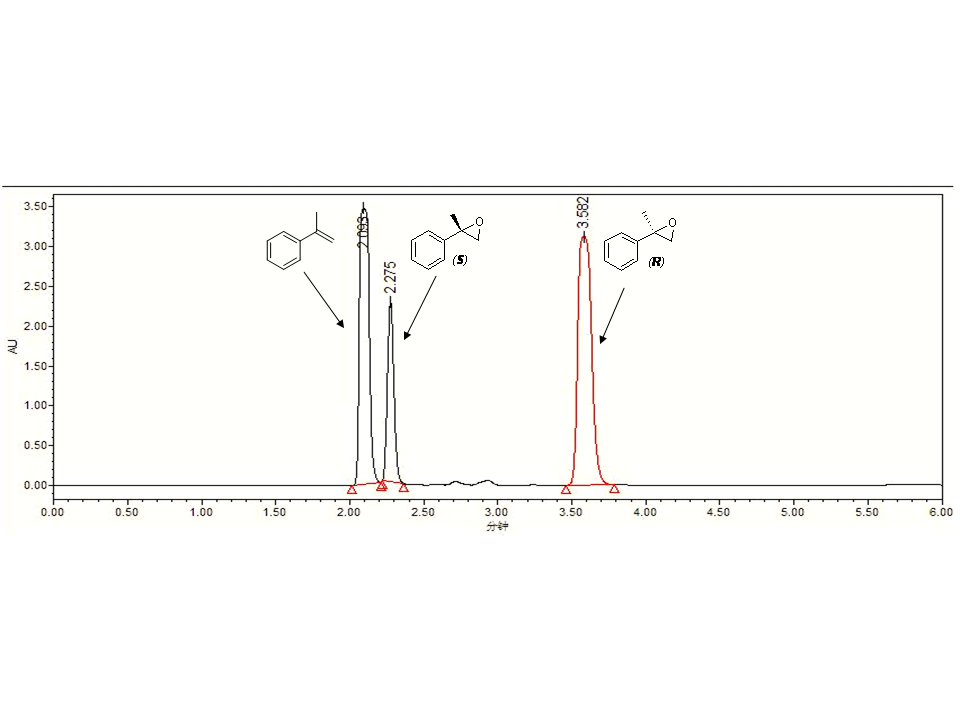


Chromatographic data:

| **Retention Time**  **(min)** | **Area**  **(μV·s)** | **% Area** | **Height**  **(μV)** | **Integration Type** |
| --- | --- | --- | --- | --- |
| 2.093 | 15,752,119 | 37.04 | 3,455,869 | bb |
| 2.275 | 6,803,909 | 16.00 | 2,255,017 | bb |
| 3.582 | 19,976,067 | 46.97 | 3,117,434 | bb |

E.e. (%) = 49% (R); Conv. (%) = 62%.

(7) Fresh circle of Entry 17, Table 6


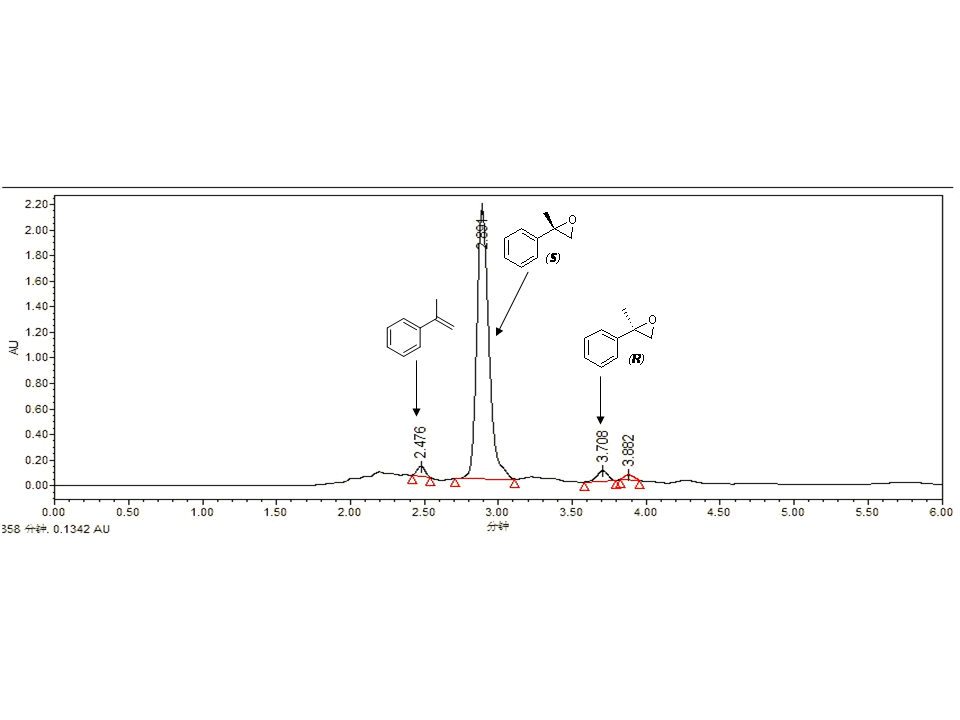


Chromatographic data:

| **Retention Time**  **(min)** | **Area**  **(μV·s)** | **% Area** | **Height**  **(μV)** | **Integration Type** |
| --- | --- | --- | --- | --- |
| 2.476 | 289,766 | 2.24 | 78,281 | bb |
| 2.891 | 12,070,271 | 93.22 | 2,108,056 | bb |
| 3.708 | 420,117 | 3.24 | 82,092 | bb |

E.e. (%) = 93% (S); Conv. (%) = 97%.

Part 3. Asymmetric epoxidation of trans-stilbene

(1) pure *trans-*stilbene


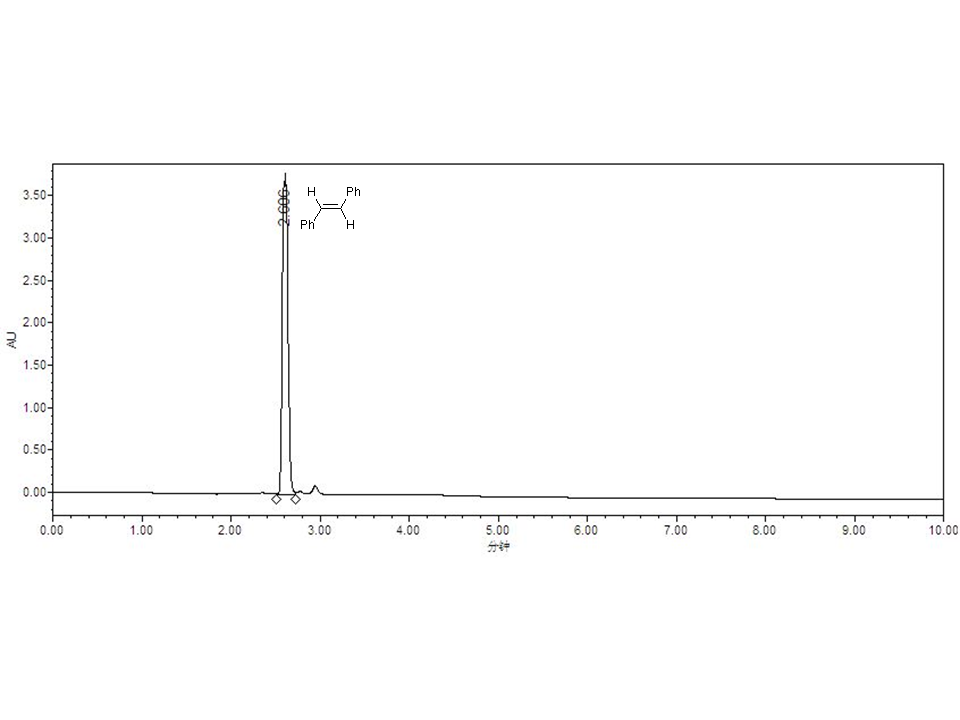


(2) racemic *trans-*stilbene oxide


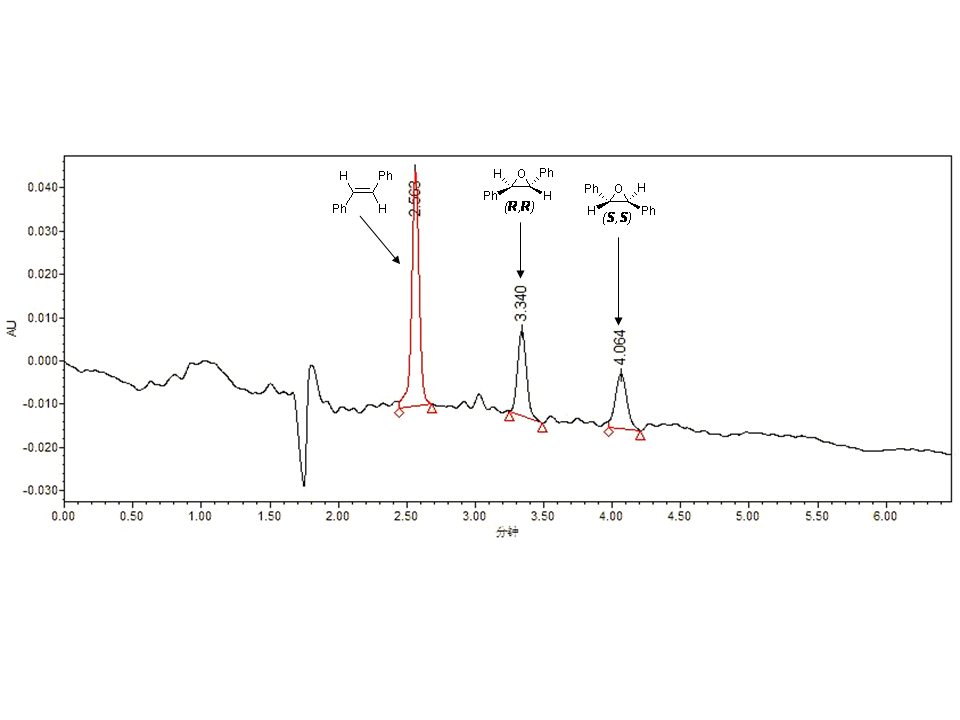


Chromatographic data:

| **Retention Time**  **(min)** | **Area**  **(μV·s)** | **% Area** | **Height**  **(μV)** | **Integration Type** |
| --- | --- | --- | --- | --- |
| 2.563 | 206,672 | 55.75 | 54,238 | VB |
| 3.340 | 89,908 | 24.25 | 19,634 | BB |
| 4.064 | 74,147 | 20.00 | 12,613 | VB |

(3) Entry 15, Table 5


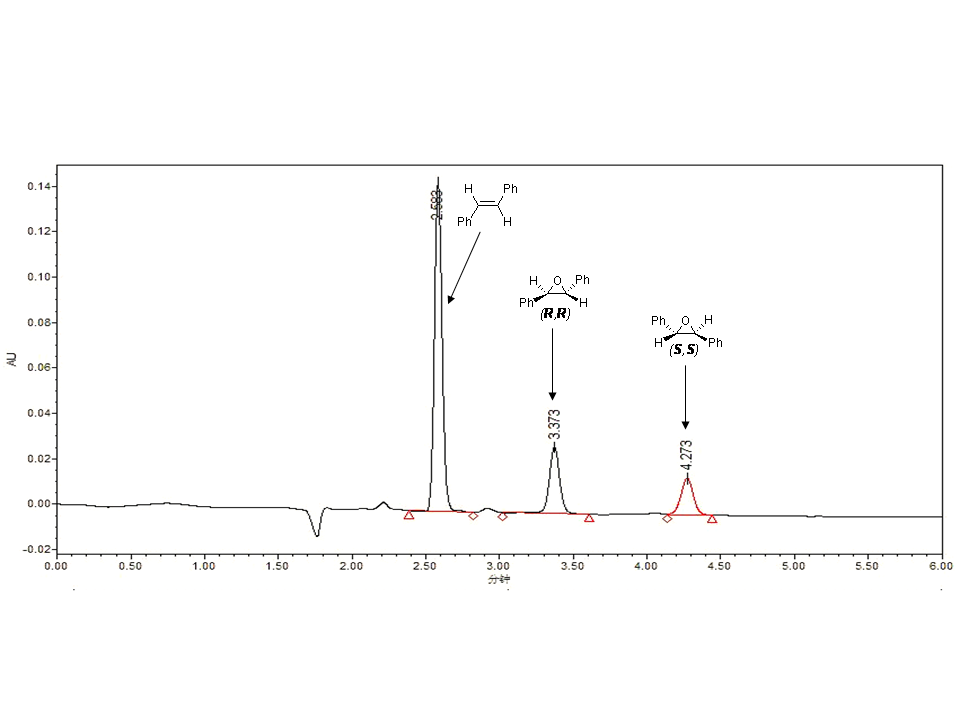


Chromatographic data:

| **Retention Time**  **(min)** | **Area**  **(μV·s)** | **% Area** | **Height**  **(μV)** | **Integration Type** |
| --- | --- | --- | --- | --- |
| 2.583 | 522,689 | 68.59 | 144,631 | bV |
| 3.373 | 144,580 | 18.97 | 29,396 | Vb |
| 4.273 | 94,812 | 12.44 | 16,176 | VB |

E.e. (%) = 20% (R,R); Conv. (%) = 31%.

(4) Entry 17, Table 5


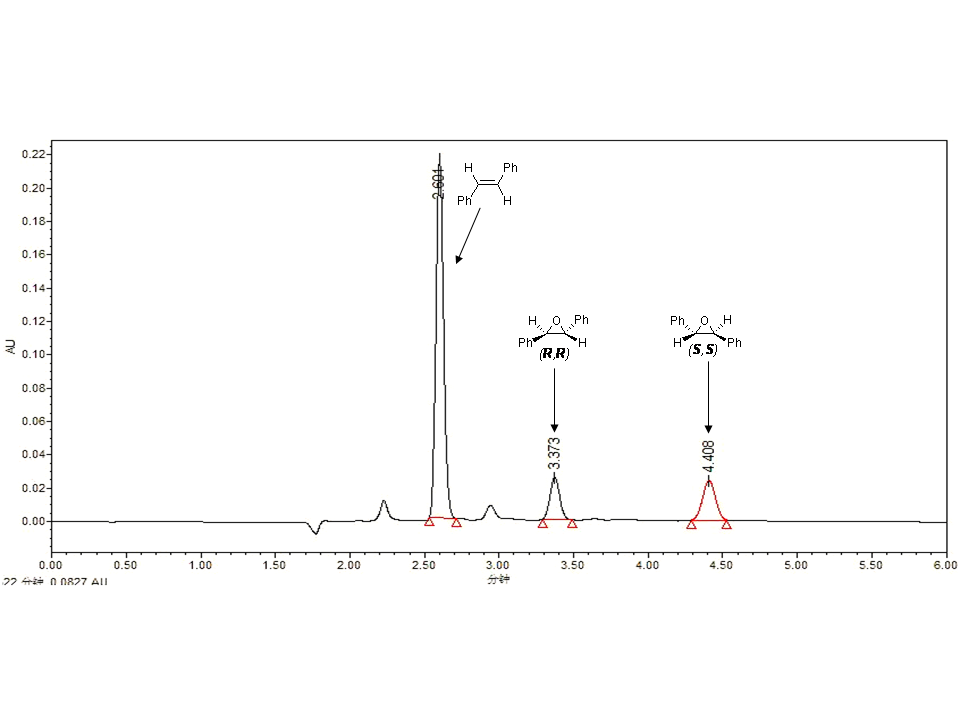


Chromatographic data:

| **Retention Time**  **(min)** | **Area**  **(μV·s)** | **% Area** | **Height**  **(μV)** | **Integration Type** |
| --- | --- | --- | --- | --- |
| 2.601 | 763,669 | 75.01 | 215,275 | bb |
| 3.373 | 112,830 | 11.08 | 25,300 | bb |
| 4.408 | 141,555 | 13.90 | 23,641 | bb |

E.e. (%) = 11% (S,S); Conv. (%) = 25%.

(5) Entry 19, Table 6


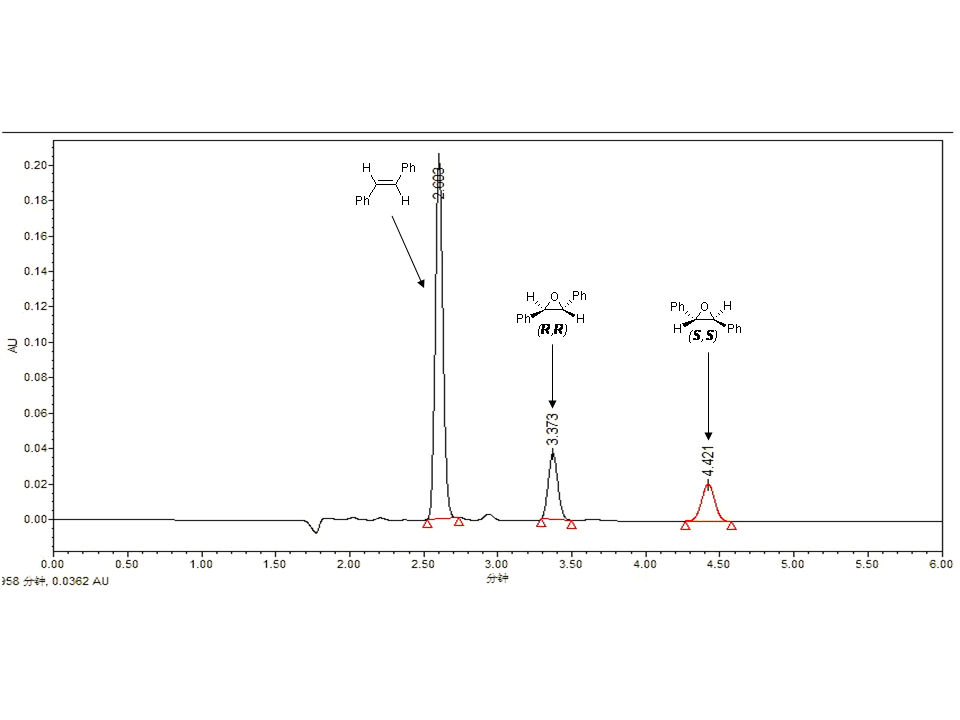


Chromatographic data:

| **Retention time**  **(min)** | **Area**  **(μV·s)** | **% Area** | **Height**  **(μV)** | **Integration type** |
| --- | --- | --- | --- | --- |
| 2.603 | 727167 | 71.08 | 202858 | bb |
| 3.373 | 166103 | 16.24 | 37118 | bb |
| 4.421 | 129729 | 12.68 | 20963 | bb |

E.e. (%) = 12% (R,R); Conv. (%) = 28%.

(6) Entry 20, Table 6


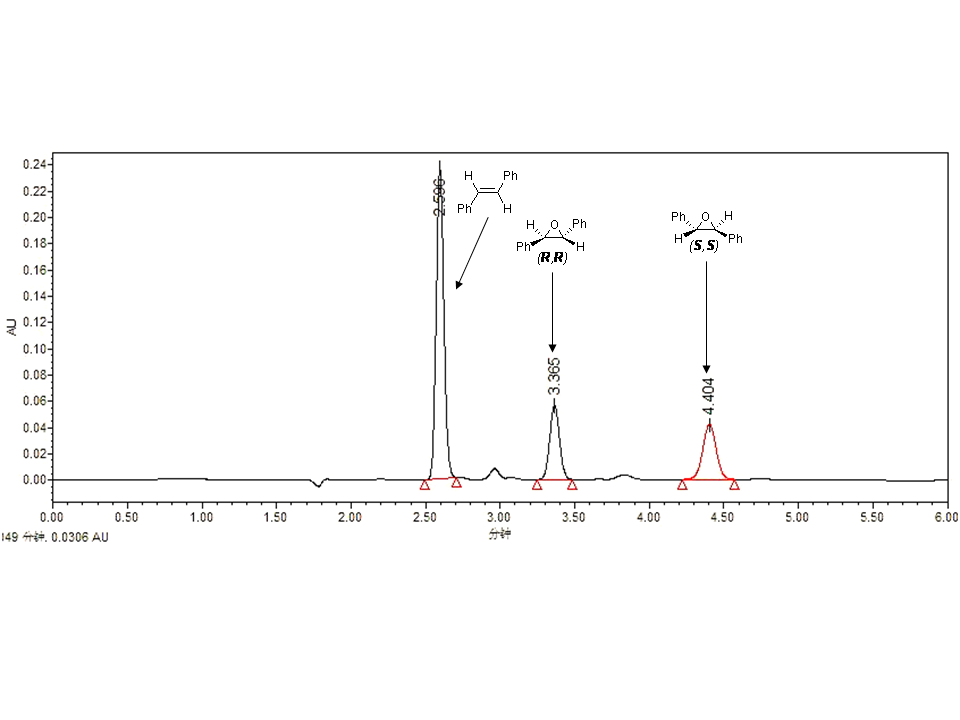


Chromatographic data:

| **Retention Time**  **(min)** | **Area**  **(μV·s)** | **% Area** | **Height**  **(μV)** | **Integration Type** |
| --- | --- | --- | --- | --- |
| 2.596 | 842,599 | 61.91 | 236,405 | bb |
| 3.365 | 257,442 | 18.91 | 56,531 | bb |
| 4.404 | 261,026 | 19.18 | 41,876 | bb |

E.e. (%) = 0.6% (S,S); Conv. (%) = 38%.

(7) Entry 25, Table 6


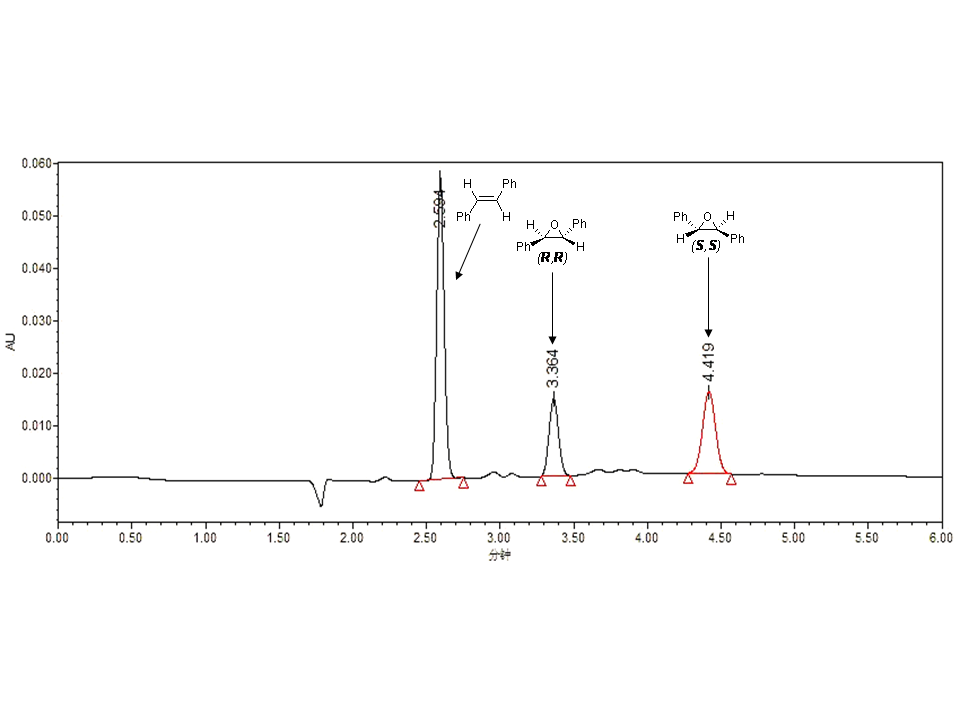


Chromatographic data:

| **Retention Time**  **(min)** | **Area**  **(μV·s)** | **% Area** | **Height**  **(μV)** | **Integration Type** |
| --- | --- | --- | --- | --- |
| 2.594 | 204,093 | 55.82 | 57,379 | bb |
| 3.364 | 65,618 | 17.95 | 14,768 | bb |
| 4.419 | 95,907 | 26.23 | 15,574 | bb |

E.e. (%) = 18% (S,S); Conv. (%) = 44%.

Part 4. Asymmetric epoxidation of indene

(1) racemic indene oxide


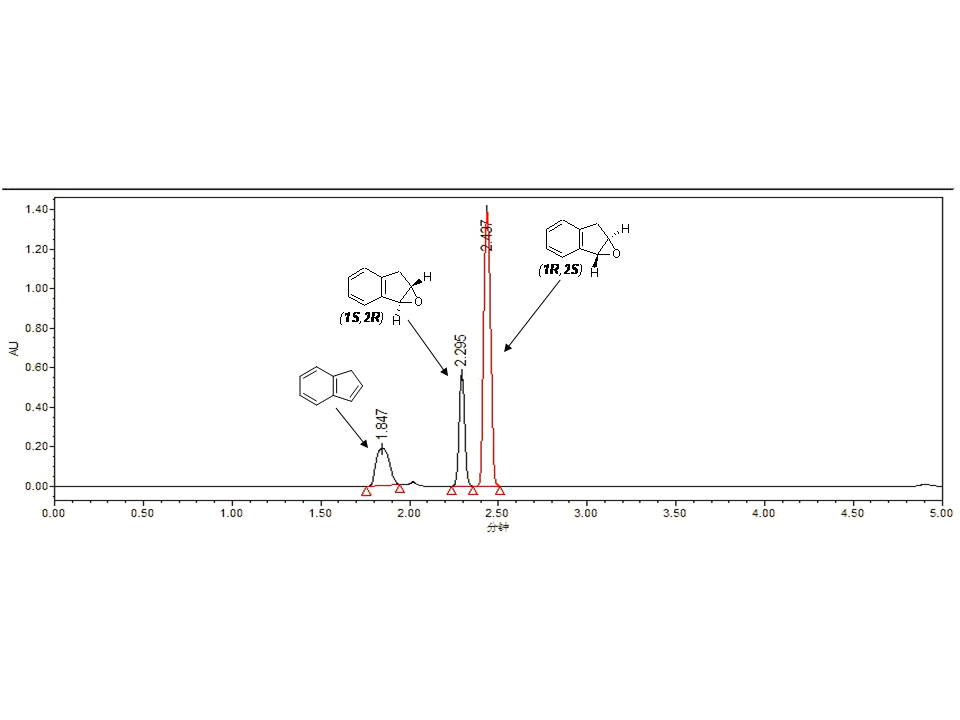


Chromatographic data:

| **Retention Time**  **(min)** | **Area**  **(μV·s)** | **% Area** | **Height**  **(μV)** | **Integration Type** |
| --- | --- | --- | --- | --- |
| 1.847 | 981,790 | 16.34 | 188,717 | bb |
| 2.295 | 1,268,573 | 21.11 | 567,699 | bb |
| 2.437 | 3,757,683 | 62.54 | 1,392,837 | bb |

(2) Entry 22, Table 5


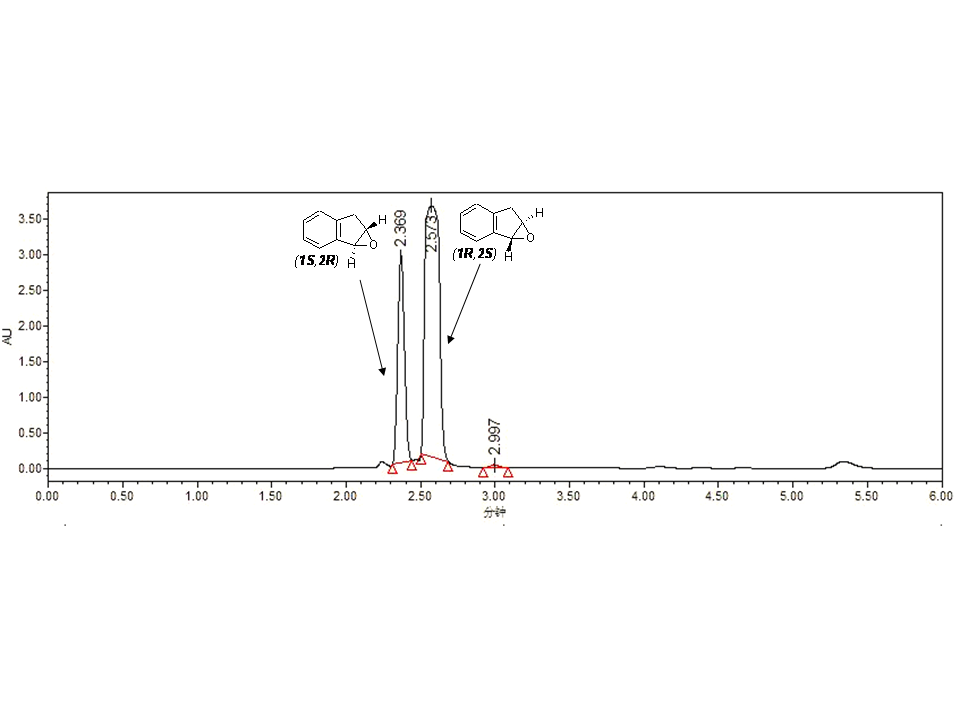


Chromatographic data:

| **Retention Time**  **(min)** | **Area**  **(μV·s)** | **% Area** | **Height**  **(μV)** | **Integration Type** |
| --- | --- | --- | --- | --- |
| 2.369 | 8,969,735 | 27.61 | 2,898,372 | bb |
| 2.573 | 23,348,105 | 71.87 | 3,521,161 | bb |

E.e. (%) = 44% (1R,2S); Conv. (%) = 100%.

(3) Entry 23, Table 5


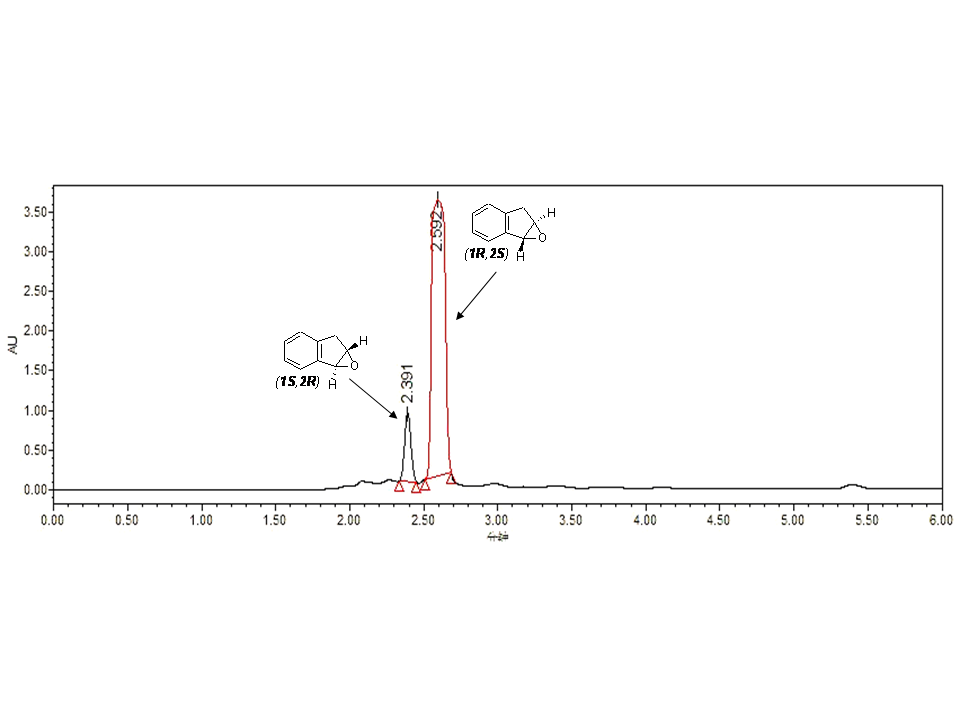


Chromatographic data:

| **Retention Time**  **(min)** | **Area**  **(μV·s)** | **% Area** | **Height**  **(μV)** | **Integration Type** |
| --- | --- | --- | --- | --- |
| 2.391 | 2,562,347 | 10.66 | 860,877 | bb |
| 2.592 | 21,466,124 | 89.34 | 3,480,927 | bb |

E.e. (%) = 78% (1R,2S); Conv. (%) = 100%.
